# In Situ Liquid‐Cell Transmission Electron Microscopy Insights Into Lithium‐Ion Battery Materials Degradation: Challenges and Emerging Solutions

**DOI:** 10.1002/advs.202521842

**Published:** 2026-01-07

**Authors:** Walid Dachraoui, Rolf Erni

**Affiliations:** ^1^ Electron Microscopy Center Empa—Swiss Federal Laboratories for Materials Science and Technology Dübendorf Switzerland; ^2^ Department of Materials ETH Zürich Zürich Switzerland

**Keywords:** battery materials, degradation, liquid cell TEM, Lithium‐ion batteries, TEM

## Abstract

Lithium‐ion batteries (LIBs) have revolutionized energy storage technologies, yet their performance, safety, and durability remain constrained by complex degradation processes that limit their ability to meet growing demands. Addressing these challenges requires a deep understanding of the underlying mechanisms. Liquid cell transmission electron microscopy (LC‐TEM), has emerged as a transformative platform for probing dynamic electrochemical processes, offering direct, multiscale insights from the crystal lattice to the full cell. Continuous advances in LC architectures, together with cutting‐edge TEM capabilities, have positioned LC‐TEM at the forefront of investigations of LIB degradation. Nevertheless, intrinsic challenges associated with liquid environments and individual cell architecture continue to limit the ability to resolve deeper details of degradation processes, creating a critical bottleneck. This review provides an overview of LIB performance challenges, recent TEM progress, and the evolution of LC‐TEM designs, emphasizing their strengths, limitations, and applications across key LIB materials. Emerging directions such as advanced microfabrication, correlative imaging, and machine learning integration are highlighted as promising pathways to expand the technique's reach and overcome current constraints. Finally, strategies are proposed to bridge in situ observations with macroscopic battery behavior. The review outlines that these emerging directions extend benefits beyond LIBs to other electrochemical and functional materials.

## Graphical abstract

Lithium‐ion battery degradation arises from complex, localized processes during operation, limiting long‐term performance. In situ electrochemical liquid cell TEM provides unique access to these mechanisms. This review summarizes degradation phenomena revealed by liquid cell TEM, traces the evolution of the three main cell designs, compares their strengths and limitations, and outlines future directions toward deeper insights into batteries and beyond.

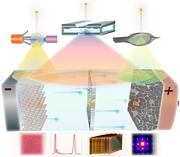

## 1 Introduction

In the era of rapid technological advancement and increasing mobile energy demands, lithium‐ion batteries (LIBs) have become a fundamental component of modern energy storage systems [1, 2, 3, 4, 5]. Their widespread use spans across numerous sectors, including portable electronics, electric vehicles (EVs), aerospace, and grid‐scale renewable energy storage [6, 7, 8]. This dominance can be attributed to their high energy density, long cycle life, and lightweight design, making them ideal for both consumer applications and large‐scale power systems [9, 10, 11, 12, 13, 14, 15]. However, the push for devices with higher power output, faster charging capabilities, and extended operational life has placed significant pressure on the existing LIB technology. As a result, the performance ceiling of current LIBs is being approached, revealing intrinsic limitations that hinder further enhancement [16, 17, 18, 19, 20].

A critical challenge lies in the progressive degradation of LIB components over time, particularly at the anode, cathode, and interfaces, as well as the electrolyte itself [21, 22, 23, 24, 25, 26, 27, 28, 29, 30]. These degradation processes, often occurring at the atomic‐ and/or nano‐scale, lead to capacity fading, reduced efficiency, increased internal resistance, and safety concerns such as thermal runaway [31, 32, 33, 34, 35]. Among the underlying factors contributing to performance decay are Li dendrite growth, solid electrolyte interphase (SEI) instability, structural transformation of electrode materials, and electrolyte decomposition (i.e., degradation) [36, 37, 38, 39, 40, 41, 42, 43, 44, 45]. These interdependent mechanisms are highly complex and dynamic, governed by cycling conditions, temperature, pressure, and intrinsic material properties [46, 47, 48, 49, 50]. Gaining a deeper understanding of the degradation pathways and developing strategies to mitigate them, is essential for advancing solutions that will enable the next generation of high‐performance batteries.

To address these challenges, researchers have employed a broad array of advanced characterization techniques aimed at revealing the structural, chemical, and electrochemical behaviors of battery materials. Traditional methods such as X‐ray diffraction [51], nuclear magnetic resonance (NMR) [52], and Raman spectroscopy [53] have provided valuable insights into bulk material properties and phase evolution [54]. However, these techniques often lack the spatial and temporal resolution necessary to observe dynamic processes at the atomic or nanoscale level. In contrast, electron microscopy and, in particular, transmission electron microscopy (TEM) have emerged as a powerful tool for directly visualizing the morphology, crystallography, and composition of battery materials with unparalleled spatial resolution [55, 56, 57, 58, 59, 60]. Recent advances in TEM instrumentation, including aberration correction, monochromated electron beams, and high‐speed direct electron detectors, have significantly enhanced the technique's ability to resolve atomic‐scale features and transient phenomena [61, 62, 63, 64, 65]. Building upon these developments, in situ TEM has become a transformative approach for studying LIBs under realistic operating conditions [66]. In situ TEM allows for real‐time observation of electrochemical reactions, structural transformations, and interfacial dynamics by integrating electrochemical functionality within the microscope environment [67, 68, 69, 70, 71, 72, 73, 74, 75]. This has enabled researchers to move beyond static imaging of post‐mortem samples toward dynamic visualization of working batteries, shedding light on processes that were previously inaccessible. A key enabler of in situ TEM for LIB research is the use of specialized liquid‐cell configurations, which mimic the battery environment by confining the electrolyte while remaining electron‐transparent for imaging [76, 77, 78, 79, 80]. Two primary types of liquid cells have been employed to date: open‐cell and closed‐cell systems. Open‐cell configurations typically use nanomanipulators to position individual nanostructures, such as nanowires, on electrodes, enabling precise control over the experimental geometry and allowing atomic‐resolution imaging [81]. These setups generally require ionic liquid electrolytes (ILEs) to withstand the high‐vacuum conditions of the TEM. In contrast, closed‐cell systems including both silicon nitride (SiN_x_)‐based liquid cells and graphene‐based liquid cells (GLCs) encapsulate the liquid environment between two electron‐transparent membranes, providing more reliable sealing [82, 83]. SiN_x_‐based LCs allow the encapsulation of conventional electrolytes and enable controlled electrochemical cycling, which facilitates real‐time observation of processes during battery operation. Notably, these designs can also incorporate flowing electrolytes, prevent depletion, mitigate electron‐beam effects on the electrolyte, and offer improved experimental control. Graphene liquid cells, on the other hand, have attracted considerable attention due to their ultrathin design and exceptional imaging resolution. The use of graphene as a membrane minimizes electron‐beam scattering while providing high mechanical strength and excellent chemical inertness, making GLCs particularly well suited for imaging sensitive electrochemical reactions.

Despite these advancements, significant challenges remain. SiN_x_‐based liquid cells, for example, offer good experimental control but are limited by spatial resolution, GLCs provide superior imaging resolution but lack robust electrochemical control, and open‐cell configurations can achieve atomic resolution, albeit under non‐realistic experimental conditions. Although each design continues to improve, these limitations represent persistent bottlenecks, highlighting the need for ongoing innovation in cell design, sample preparation, and strategies to bridge the gap between in situ observations and real‐world battery operation.

Numerous reviews have addressed in situ TEM for batteries, focusing either on methodology or on experimental results [84, 85, 86, 87, 88, 89, 90]. Here, we provide a comprehensive overview of LIB degradation processes, recent TEM advancements including the latest LC designs and their applications, and the key bottlenecks that remain. Unlike previous reviews, our work uniquely integrates degradation mechanisms, methodological advances, and practical insights from LC‐TEM, thereby providing a holistic framework that connects nanoscale observations to macroscopic battery behavior. We first outline critical degradation mechanisms at the anode, cathode, electrolyte, and interfaces, encompassing structural, chemical, and mechanical changes. We then review state‐of‐the‐art TEM capabilities and the evolution of LC‐TEM techniques, highlighting recent developments. Special emphasis is placed on LC configurations, open‐cell, closed‐cell, and graphene‐based detailing, their strengths, limitations, and contributions to understanding LIB behavior at the nanoscale. We further summarize the most impactful insights gained from each configuration and propose strategies to overcome current limitations, including advanced microfabrication, improved electrolyte compatibility, and hybrid imaging platforms. By addressing both capabilities and challenges, this review charts a pathway toward more accurate and predictive characterization of next‐generation LIB materials and interfaces. Importantly, we also discuss approaches to bridge nanoscale insights from LC‐TEM with the electrochemical behavior of full‐scale batteries, thereby enhancing the relevance of in situ TEM beyond model systems. Finally, we outline how the strategies presented here could extend the benefits of LC‐TEM to other electrochemical and functional materials, offering broader opportunities for materials discovery and design.

## 2 History of LIBs and the Actual Performance Challenges

**FIGURE 1:**
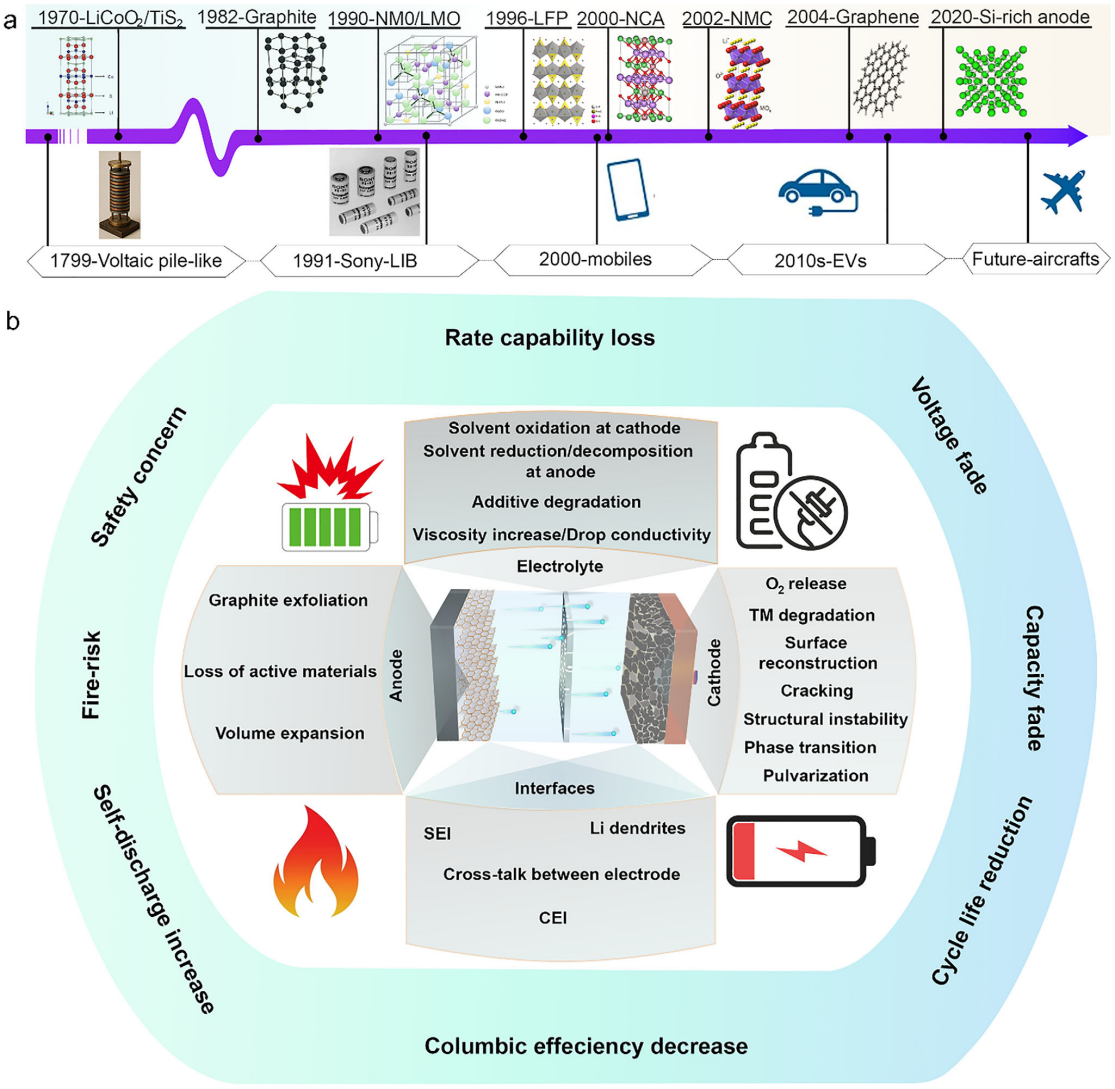
(a) Historical evolution of LIBs. (b) Main degradation processes and their consequences on LIB performance.

Battery technology has evolved since 1799, when Alessandro Volta introduced the voltaic pile, the first prototype of a modern battery (Figure 1) [91]. However, it wasn't until the 1970s that the focus shifted decisively toward lithium‐based chemistries, marking a pivotal transformation in electrochemical energy storage. The discoveries of titanium disulfide (TiS_2_) and lithium cobalt oxide (LiCoO_2_: LCO) as cathode materials laid the foundation for rechargeable LIBs [92, 93]. In 1982, the introduction of graphite as an anode capable of reversibly intercalating Li‐ions significantly enhanced reversibility and cycle life [94]. These breakthroughs culminated in the commercialization of the first LIB by Sony in 1991, which revolutionized portable electronics. In the early days of lithium‐ion batteries, progress was often hindered by the lack of stable, high‐performance electrolytes. Researchers, therefore, concentrated on developing electrolytes with both high ionic conductivity and chemical stability [95]. At the same time, efforts were directed toward high‐voltage and high‐capacity cathode materials. By the 1980s and early 2000s, several new cathode systems had emerged, overcoming many of the limitations of earlier chemistries [96, 97]. Over the past few decades, LIB cathodes have evolved remarkably (Figure 1a). LiMn_2_O_4_ (LMO), developed in the mid‐1980s and commercially introduced in the early 1990s, features a spinel structure with 3D lithium pathways [96]. It delivers moderate capacity (∼120 mAh/g) and excellent thermal stability, though high temperatures can lead to capacity fading due to manganese dissolution. LiNi_0.5_Mn_1.5_O_4_ (NMO), introduced in 1990, is a high‐voltage spinel (∼4.7 V) that offers impressive energy density, but its stability must be carefully managed [97]. LiFePO_4_ (LFP), developed in 1996, uses an olivine structure with 1D lithium channels, providing outstanding safety, long cycle life, and chemical stability, although at a slightly lower voltage (∼3.4 V) [98]. LiNi_0.33_Mn_0.33_Co_0.33_O_2_ (NMC111), developed in 2002, strikes a balance between energy density, safety, and cost, becoming a popular choice for electric vehicles [99]. Finally, LiNi_0.8_Co_0.15_Al_0.05_O_2_ (NCA), developed in the early 2000s and commercialized by Panasonic and Tesla, is a nickel‐rich layered cathode that delivers high energy density for long‐range EVs, though careful battery management is needed to ensure thermal stability [100]. Together, these cathode materials reflect the continuous effort to optimize LIBs for performance, safety, and cost. Since the 2010s, high‐voltage spinels and lithium‐rich layered oxides have attracted significant attention as next‐generation cathodes. Interestingly, around 2010, graphene began to revolutionize anode materials for LIBs, while silicon‐based anodes started emerging in the early 2020s [101, 102].

Despite these advances, the performance of LIBs has gradually approached intrinsic limits due to a range of complex and interrelated degradation processes affecting the electrodes, electrolyte, and interfaces (Figure 1b) [47, 102, 103, 104, 105]. At the cathode, a complex degradation involves cracks, phase transition, oxygen evolution, surface reconstruction, cation mixing, and lattice collapse, leading to voltage fade, capacity loss, and reduced ionic and electronic conductivity (i.e., particularly in high‐energy Ni‐rich materials such as LiNi_0.8_Mn_0.1_Co_0.1_O_2_ (NMC811) or NCA) [106]. Particle cracking caused by repeated cycling induces mechanical disconnection and exposes fresh surfaces to the electrolyte, which exacerbates electrolyte oxidation and cathode electrolyte interphase (CEI) formation [106, 107, 108, 109]. Transition metal dissolution is another key degradation pathway, where metals like Ni, Co, or Mn dissolve into the electrolyte and migrate to the anode, contributing to cross‐talk and parasitic reactions [110]. At the anode, graphite can suffer from exfoliation, Li plating, and SEI instability under high current or low‐temperature conditions [111, 112, 113, 114]. For high‐capacity materials such as silicon or Li metal, severe volume expansion during lithiation/delithiation cycles leads to particle pulverization, contact loss, and repetitive SEI breakdown, which consumes electrolyte and cyclable lithium and promotes dead Li (i.e., inactive Li) formation [115, 116, 117, 118]. In extreme conditions, dendritic Li can grow and cause short circuits, posing serious safety risks [119, 120, 121].

The electrolyte, while essential for ionic transport, is inherently unstable at both high voltage and low potential. At the cathode, oxidative decomposition of solvents and salts (e.g., LiPF_6_) produces resistive films, gases, and reactive species such as hydrofluoric acid (HF), which attack both electrodes and accelerate transition metal dissolution. At the anode, reductive decomposition forms SEI but also leads to gas generation, viscosity increase, and loss of ion mobility. Electrolyte additives, although introduced to stabilize interfaces, can themselves degrade and trigger undesired side reactions, especially at elevated temperatures or voltages [122, 123, 124].

At the interfaces, the stability of SEI and CEI layers is central to long‐term performance. A stable SEI protects the anode but often becomes unstable under cycling, especially with silicon or Li‐metal anodes, leading to continuous electrolyte consumption and impedance rise [125, 126]. Similarly, the CEI on the cathode surface, particularly in Ni‐rich systems, is prone to thickening and increased resistance, resulting from electrolyte oxidation and lattice distortion at the cathode surface [127]. Furthermore, crosstalk between electrodes, involving the migration of metal ions and soluble species, links interfacial degradation across the entire cell [128]. These intertwined processes collectively result in capacity fading, voltage drop, poor rate capability, self‐discharge, and reduced cycle life [129].

Altogether, the current generation of LIBs, though mature and commercially dominant, faces significant performance and stability limitations due to fundamental degradation pathways, which restrict their application in emerging technologies such as aircraft. This highlights the need for a thorough understanding of each process at the atomic scale and the intricate interplay between them in order to develop effective solutions.

## 3 Cutting‐Edge TEM Techniques for Advanced Materials Characterization

**FIGURE 2:**
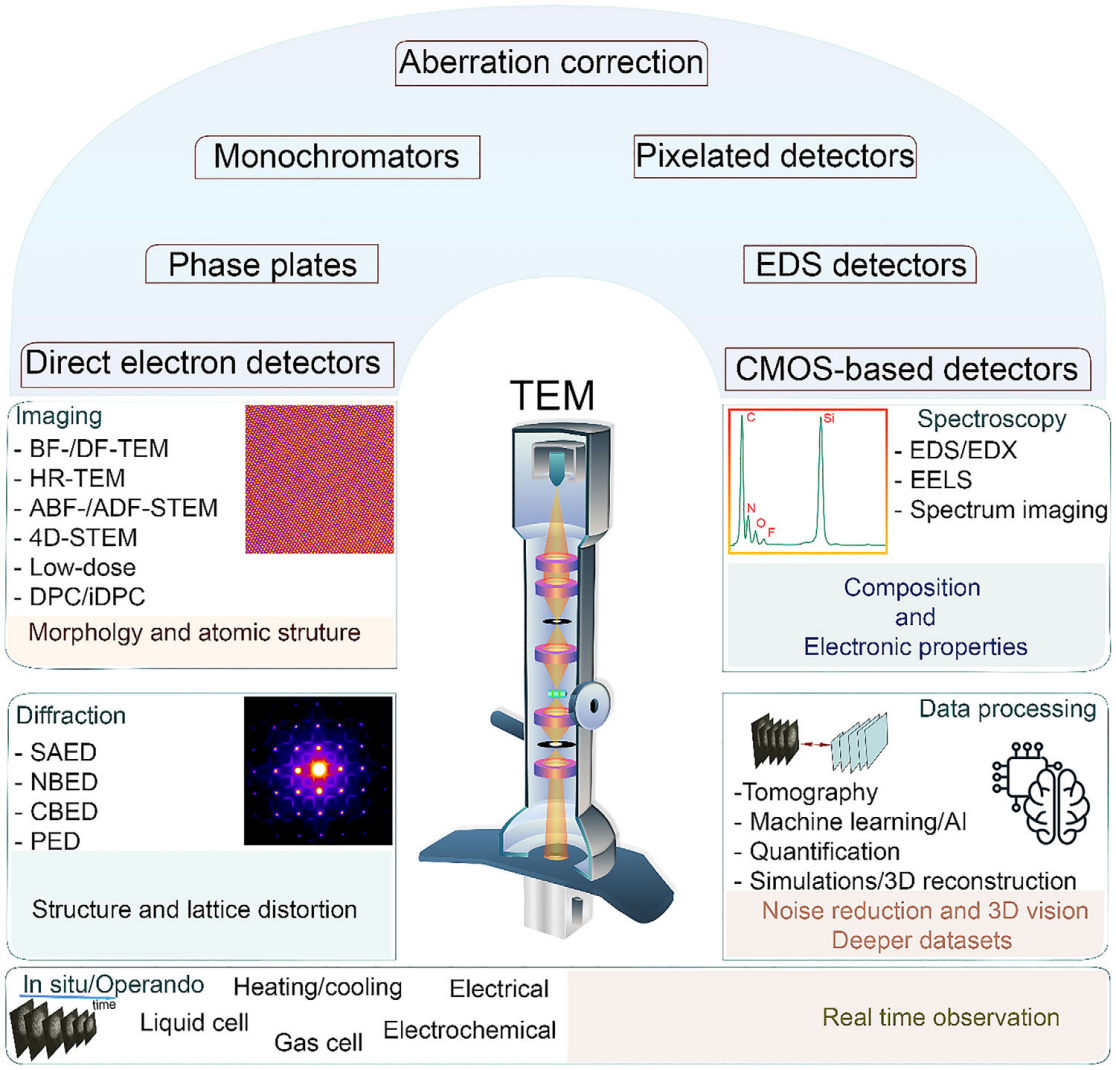
Schematic illustration of the TEM and its capabilities: The main equipment and different operational techniques.

Transmission electron microscopy has become an indispensable and highly versatile tool for characterizing materials across multiple length scales. Since its invention in the 1930s, TEM has undergone continuous and transformative advancements that have greatly expanded its imaging and analytical capabilities [130]. A pivotal milestone was achieved end of the 1990s with the development and implementation of aberration‐corrected optics, which dramatically improved spatial resolution and enabled routine atomic‐resolution imaging [131]. Modern TEM instruments now allow for the direct visualization of individual atomic columns, even in structurally complex and compositionally heterogeneous materials. Over the past decades, further innovations in TEM hardware have pushed the boundaries of nanoscale analysis, particularly through the integration of advanced analytical techniques. Aside from electron energy‐loss spectroscopy (EELS), which benefits from new detectors and monochromatic electron sources, energy‐dispersive X‐ray spectroscopy (EDX) has seen significant improvements, with modern systems employing large solid‐angle silicon drift detectors arranged in multi‐detector geometries [132]. These configurations substantially increase X‐ray collection efficiency and enable atomic‐scale elemental mapping, even in beam‐sensitive materials. When combined with aberration‐corrected scanning TEM (STEM) imaging, this results in chemical analysis with high spatial resolution. In parallel, the development of precession electron diffraction (PED) has enhanced the accuracy of crystallographic analysis by minimizing dynamical scattering effects and improving orientation mapping and phase identification, particularly in nanocrystalline and complex systems [133]. When coupled with fast, high‐sensitivity detectors and synchronized acquisition systems, PED allows for quantitative and orientation‐resolved diffraction analysis. Collectively, these advancements in spectroscopy and diffraction techniques have extended the capabilities of TEM well beyond conventional imaging, enabling comprehensive structural and chemical characterization at the nanoscale. TEM is now widely employed in materials science, notably in energy research and battery development, where understanding structure–property relationships at the atomic level is essential for optimizing electrochemical performance. Its versatility stems from a unique combination of multimodal capabilities, including imaging, electron diffraction, spectroscopy, and increasingly sophisticated data processing and computational analysis (Figure 2).

Imaging modes offer direct insights into morphology, crystal structure, and atomic configuration of battery materials across length scales, from the micrometer to the atomic level. Bright field (BF) and dark field (DF) TEM, high‐resolution TEM (HRTEM), and high‐angle annular dark field STEM (HAADF‐STEM), along with other STEM modalities are powerful for resolving defects, phase boundaries, and degradation mechanisms, with HAADF‐STEM providing contrast sensitive to atomic number (Z), thickness, and mass. Annular bright field STEM (ABF‐STEM) is particularly valuable for detecting light elements such as lithium and oxygen, enabling visualization of Li‐ion pathways and low‐Z structural motifs. Furthermore, differential phase contrast (DPC) and integrated DPC (iDPC) imaging, especially when combined with 4D‐STEM or segmented detectors, enable quantitative mapping of electrostatic potentials and local electric fields. These methods are increasingly applied to probe internal fields at solid–solid interfaces (e.g., grain boundaries), polarization effects, and ion migration processes in complex battery architectures [134, 135]. While imaging reveals atomic and structural details, electron diffraction techniques provide precise crystallographic information from localized regions of (battery) materials complementing the imaging mode. Selected area electron diffraction is widely used to probe structural properties and lattice distortions in cathode materials, averaging over several tens to hundreds of nanometers. Nano‐beam diffraction enables phase and orientation mapping at the nanoscale, offering insights into grain boundaries, domain evolution, and fracture behavior in electrode particles. Convergent beam electron diffraction, on the other hand, is particularly powerful for detecting local lattice distortions, symmetry breaking, and internal strain field phenomena highly relevant for materials prone to oxygen evolution and surface reconstruction under electrochemical stress (Figure 2) [136]. Beyond structural characterization, spectroscopy techniques add a chemical and electronic dimension to TEM analysis. EDX spectroscopy and EELS provide complementary information at sub‐nanometer‐scale resolution. EDX is well‐suited for elemental mapping and quantitative analysis, while EELS grants access to electronic fine structure, oxidation states, and local bonding environments. These capabilities are especially critical for tracking redox processes in cathode materials, characterizing the chemical composition of the SEI/CEI, and probing localized valence changes during electrochemical cycling [137, 138]. Finally, advances in data processing are transforming TEM into a truly quantitative and predictive platform. Electron tomography addresses the inherent limitation of 2D projections by enabling 3D reconstruction of complex morphologies and architectures. More recently, the integration of machine learning (ML) has emerged as a powerful approach to enhance TEM data analysis and increase acquisition speeds in dynamic measurements. ML enables automated recognition, segmentation, and classification of structural features such as phase boundaries, defects, heterogeneities, and dynamic transformation fronts, thereby reducing manual bias and accelerating interpretation. Beyond feature extraction, ML can uncover hidden correlations across large datasets, enabling statistical analysis and predictive modeling of structural and chemical evolution under varying conditions. Combined with advanced simulations and 3D modeling, these approaches elevate TEM from a descriptive imaging tool to a data‐driven framework for discovering, designing, and optimizing complex functional materials systems and next‐generation battery materials in particular [139, 140].

In parallel, advanced in situ TEM techniques have been developed with a variety of sample cell configurations, enabling the study of materials under realistic environmental conditions. These include controlled atmospheres (gas or liquid), temperature variations (heating and cooling), and external stimuli such as electrical biasing or electrochemical operation, allowing researchers to closely mimic real‐world device conditions during observation [141].

## 4 Progress in the Design of TEM Liquid Cell Configurations for the Study of LIB Materials

**FIGURE 3:**
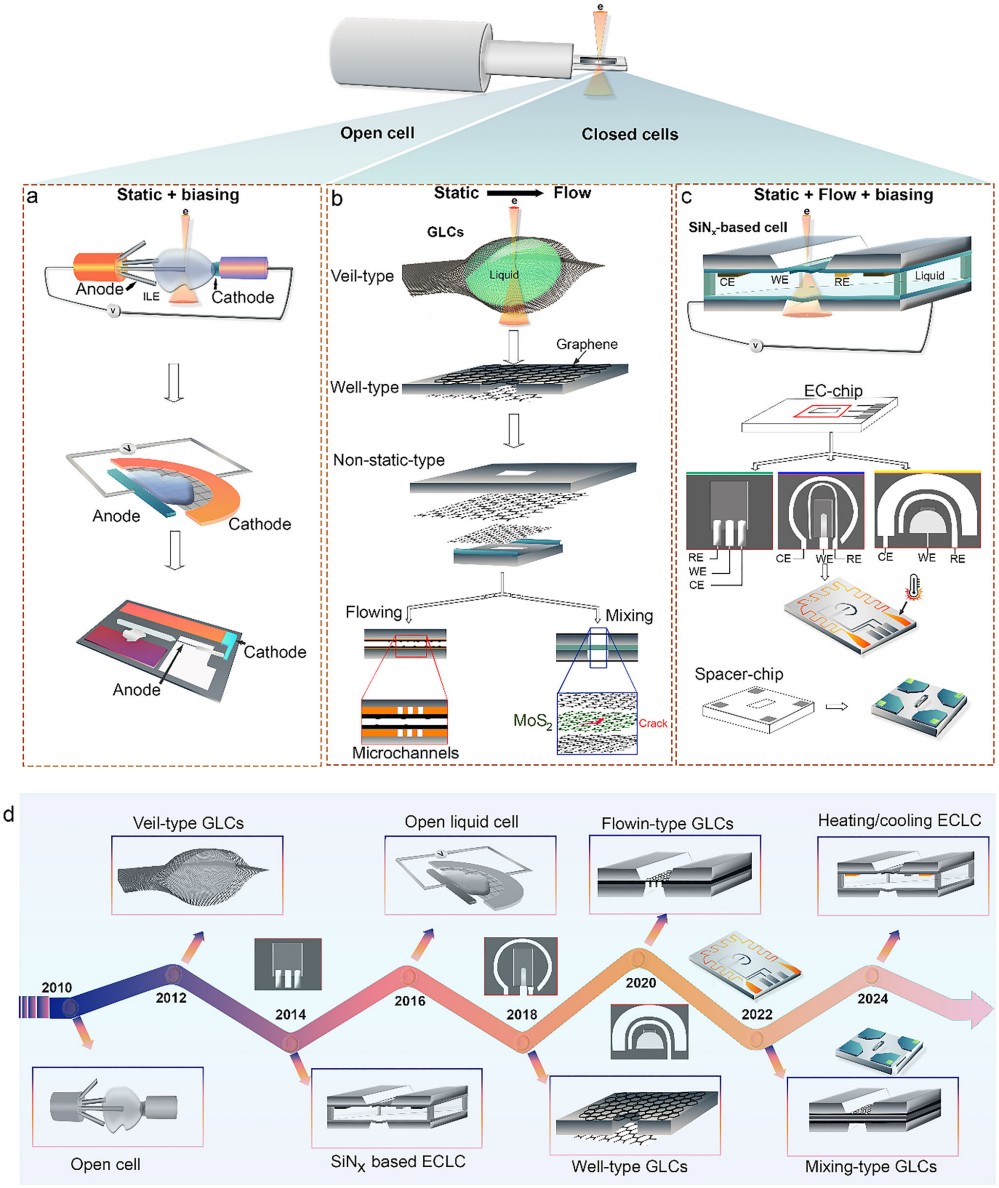
Evolution and configurations of liquid cell designs for in situ TEM studies. (a–c) Schematic illustration of major liquid cell configurations, divided into open cells and closed cells. Open cells feature an exposed ionic liquid between nanoscale electrodes. Closed cells include GLCs and SiN_x_​‐based liquid cells. (d) Timeline showing the development of liquid cell designs from the first open cell in 2010.

In situ TEM has become an indispensable technique in LIB research, largely driven by the development of advanced LC configurations that enable real‐time visualization of electrochemical processes. These approaches allow researchers to monitor the dynamic evolution of key components, including the cathode, anode materials, and interfaces, under conditions that closely mimic real battery operation, such as organic electrolytes and electrochemical cycling. The core principle of in situ LC‐TEM is its ability to directly observe material transformations in liquid environments within the high‐vacuum TEM setting, made possible by various functional cell designs. Among these, two main categories have emerged: open liquid cells and closed liquid cells. Closed liquid cell, including G‐based and SiN_x_‐based designs, offer unique advantages and have undergone significant improvements in recent years. Figure 3 illustrates the schematics and evolution of these three types of cell configurations, highlighting their progression from initial implementations to state‐of‐the‐art designs and commercial solutions in some cases.

### 4.1 Open Liquid Cell

The nanoscale open‐cell configuration for LIB studies in TEM was first introduced by Huang et al. in 2010 (Figure 3a,d), representing a significant breakthrough in real‐time electrochemical characterization at the nanoscale [142]. As illustrated in Figure 3a, the core concept of this open‐cell nanobattery involves using a TEM nanomanipulator to precisely position individual nanostructures, such as nanowires and nanofiber materials, onto an electrode. This setup enables direct atomic‐scale visualization of dynamic morphological changes during lithiation and delithiation, providing critical insights into anode and cathode behavior. In their original demonstration, Huang et al. proposed the use of a droplet of ILE placed on a LiCoO_2_ cathode [142]. The molten salt nature of ILEs, characterized by their very low vapor pressure, enables them to withstand the high vacuum conditions (∼10^−5^ Pa) of the TEM while still maintaining effective Li‐ion transport between electrodes [143]. This ILE‐based open‐cell system proved especially well‐suited for 1D nanostructures, where materials like nanowires or nanotubes could be partially immersed in the electrolyte. In this configuration, these structures serve both as electrochemically active components and as electrical contacts, making the setup ideal for probing their behavior under operando conditions. More recently, a new generation of open‐cell designs has been developed to further improve flexibility and compatibility with multidimensional materials. This involves the use of a TEM grid as a platform to support the active material (e.g., nanoparticles) along with the ionic liquid, and the integration of a specially designed anode. This evolution significantly broadens the applicability of open‐cell TEM setups, enabling the study of more complex material systems and geometries while maintaining high spatial and temporal resolution [143]. A notable example of these advances is the work by Zhang et al., where an ILE‐based open‐cell configuration was developed to more closely resemble the architecture of a real battery. In this design, Li_4_Ti_5_O_12_ (LTO) nanoparticles were deposited onto a carbon film acting as the working electrode, while a Li‐metal counter electrode was immersed in the ionic liquid electrolyte. This setup enabled operando EELS mapping of Li occupancy and transport within the LTO spinel structure during charge/discharge cycling, providing unprecedented insight into Li‐ion migration pathways and local redox dynamics [144].

Strengths: The open‐cell configuration offers several advantages that make it a powerful tool for investigating LIB materials at the nanoscale. Its primary strength is the ability to achieve atomic‐scale resolution under operando conditions, enabling real‐time observation of structural and morphological changes in individual nanostructures such as nanowires and nanotubes. Furthermore, the use of ILEs ensures vacuum compatibility due to their extremely low vapor pressure, preventing rapid evaporation under the high vacuum of the TEM and eliminating the need for fragile membranes. Compared to more complex sealed liquid cells, this setup is relatively simple, cost‐effective, and facilitates straightforward electrical biasing and mechanical manipulation using nanoprobes.

### 4.2 Graphene Liquid Cells

GLCs represent a major advancement in in situ TEM for imaging materials in real time such as studying electrochemical processes in LIB materials (Figure 3b). First introduced around 2011 (Figure 3d), GLCs employ atomically thin, or few‐layer graphene sheets as both encapsulation membranes and, in some cases, conductive supports [145]. Their development was motivated by the need for ultra‐thin, electron‐transparent, and mechanically robust enclosures capable of containing liquid under the high‐vacuum conditions of TEM. The design of GLCs has evolved significantly. Early efforts focused on the veil‐type configuration, in which a thin liquid layer was sandwiched between two graphene sheets and held in place by van der Waals forces (Figure 3b). This approach provided exceptional spatial resolution owing to the minimal liquid thickness but was prone to rapid liquid evaporation and lacked well‐defined geometry, limiting its suitability for extended experimental measurements. Veil‐type GLCs have been extensively used since their introduction to explore materials behavior in liquids at the atomic scale, revealing mechanisms that were previously inaccessible. Dachraoui et al. employed this design to study the nucleation of Pt nanoparticles, capturing their evolution from single atoms to mature nanoparticles and uncovering a complex multistep pathway [146]. In a follow‐up study, the same group demonstrated the atomic‐scale growth mechanism of Pt–Pd bimetallic nanoparticles, highlighting the intricate interplay between alloying, coalescence, and surface atom rearrangements [147]. Recently, Dong et al. have investigated this liquid cell type to shed light on the dissolution‐regrowth mechanism dominating the shape evolution of silver NPs [148].

To overcome the limitations of the veil‐type design, the well‐type GLC was developed (Figure 3b). In this configuration, a SiN_x​_ membrane with predefined holes serves as a liquid reservoir, which is sealed on both sides with graphene sheets to create a confined yet stable liquid environment. This architecture provides improved volume control and mechanical stability while maintaining high electron transparency. The holey SiN_x_ support further facilitates device integration and precise positioning within the TEM. Importantly, it also enables the fabrication of a controlled number of liquid cells, thereby enhancing reproducibility and experimental reliability [149]. However, well‐type GLCs still lack liquid flow, a critical feature for certain applications such as in situ studies of LIB materials where continuous electrolyte flow is necessary to sustain ion transport, minimize concentration gradients, and avoid electrolyte degradation. Well‐type GLCs have been widely adopted to elucidate complex behaviors of nanomaterials in their native liquid environments. Lim et al. performed a statistical analysis of gold nanoparticle growth and motion, revealing dynamic pathways and population‐level trends inaccessible by conventional TEM [150]. This cell type has also enabled quantitative insights into nanoparticle coalescence, demonstrating how surface ligands modulate interparticle interactions and controlling the growth and structural evolution of nanoparticles [151].

Building upon these advances, researchers developed the liquid‐flowing‐type GLC (Figure 3b), which integrates microfluidic channels to enable continuous or pulsed liquid flow during imaging. These channels are typically sealed by graphene membranes, providing a flexible and vacuum‐compatible enclosure [146]. By allowing replenishment of the liquid phase and removal of reaction byproducts, the flow‐type GLC improves chemical fidelity and extends the duration of in situ experiments, thereby addressing key limitations of static liquid cells. Leveraging controlled liquid supply, flow‐type GLCs facilitate the study of nanoscale processes under well‐defined and continuously refreshed reaction conditions. Dunn et al. investigated dynamic behaviors of Au nanoparticles by separately injecting water and Au nanoparticle solution, enabling direct tracking of nanoparticle motion paths with high precision. Beyond motion dynamics, this configuration has enabled the study of uranyl acetate crystallization and growth, achieved by mixing uranyl acetate with a phosphate buffer. Other studies have shown that the continuous flow itself can drive nanoparticle motion and has allowed operando visualization of etching mechanisms at the nanoscale [152].

The most recent innovation is the mixing‐type GLC (Figure 3b), designed to enable real‐time mixing of different liquids or reactants during TEM observation. In this architecture, two separate microchip platforms support graphene membranes, with liquids initially kept isolated. Controlled mixing is triggered by rupturing a thin MoS_2_ membrane that serves as a breakable barrier between the compartments [153]. This configuration opens new opportunities for investigating dynamic processes such as interfacial reactions, solution chemistry, or nanoscale transformation kinetics under well‐controlled, stimulus‐responsive conditions. Mixing‐type GLCs have proven particularly powerful for probing liquid‐phase chemical reactions with high spatial and temporal resolution. By mixing two precursor solutions within the cell, Kelly et al. captured the multistep formation pathway of calcium carbonate (CaCO_3_) in real time with sub‐second temporal resolution. The precise control over reaction initiation provided by this design has also advanced understanding of degradation mechanisms in quantum‐sized semiconductor nanocrystals. A recent study revealed the degradation pathways of CdS nanocrystals by directly tracking their structural transformations during water exposure in real time [154].

Taken together, these successive GLC designs represent a clear progression from simple static systems to increasingly sophisticated, functional platforms. They have substantially enhanced the realism and controllability of nanoscale investigations, broadening the scope of in situ liquid‐phase TEM studies across diverse fields of materials science including LIB materials.

Strengths: GLCs offer several key advantages for nanoscale studies. Their atomically thin graphene membranes provide exceptional spatial resolution, enabling direct observation of structural and interfacial changes at the atomic scale. The high electrical and thermal conductivity of graphene further helps dissipate heat and minimize beam‐induced damage, making GLCs particularly suitable for imaging beam‐sensitive processes. The development of non‐static GLC designs has advanced this platform beyond the limitations of traditional static modes: flow‐type GLCs enable continuous replenishment of the liquid phase, reducing depletion effects and allowing longer investigations of dynamics. The mixing‐type configuration further broadens experimental capabilities by enabling controlled mixing of precursors to initiate processes and monitor their evolution at the atomic scale. The advantages of GLCs stem from the unique properties of graphene, making membrane selection crucial for LP‐TEM performance. Like an aquarium window, LP‐TEM windows must be highly transparent and mechanically stable, but they also require electron transparency and sealing capability under TEM high‐vacuum conditions. Graphene's atomic thickness (3.4 Å) and low atomic number (*Z* = 6) provide exceptional electron transparency, yielding vacuum‐like contrast and minimal energy loss [145]. This suppresses multiple scattering, enabling clearer chemical analyses via EELS or EDS. Combining atomic‐resolution imaging with chemical identification, GLC‐TEM offers powerful insights into liquid‐phase phenomena.

### 4.3 SiN_x_‐Based Closed Liquid Cell

While open liquid cells (Section 4.1) have enabled initial explorations of electrochemical processes, and graphene‐based liquid cells (Section 4.2) have pushed the limits of spatial resolution, both systems present limitations when it comes to replicating the realistic operational conditions of LIBs (Figure 3c). In contrast, SiN_x_‐based closed liquid cells provide a more robust and integrated platform that closely mimics the actual architecture and environment of commercial batteries. This is primarily due to their ability to incorporate real liquid electrolytes in a sealed, vacuum‐compatible system with integrated multi‐electrode configurations. This advanced approach utilizes a microfabricated closed liquid cell in which SiN_x_ membranes act both as the sealing layers and as electron‐transparent imaging windows (Figure 3c) [155]. These membranes enclose the liquid (electrolyte) while maintaining compatibility with high‐energy electron beams, enabling real‐time observation of electrochemical processes under operando conditions. This configuration allows for precise in situ electrochemical testing inside the TEM. The system can be operated in either static mode, where the liquid remains confined or in flow mode, which is particularly advantageous to continuously renew the electrolyte and eliminate gas bubbles formed during cycling. The flow mode ensures more stable electrochemical conditions by preventing local electrolyte depletion and maintaining a homogeneous environment. A critical aspect of this platform is the integration of a three‐electrode configuration within the MEMS chip, enabling accurate and quantitative in situ electrochemical control. This configuration includes a working electrode (WE), a reference electrode (RE), and a counter electrode (CE) (Figure 3c) [155]. The WE, typically composed of the active battery material under investigation, is the site where the electrochemical reactions occur and is directly positioned within the imaging area for correlative analysis. The RE serves as a stable potential baseline, allowing precise control and monitoring of the potential applied to the working electrode. Meanwhile, the CE completes the electrochemical circuit and is designed to accommodate the required current without becoming a limiting factor in the system. This configuration ensures reproducible and stable electrochemical conditions, which are essential for capturing dynamic transformations and degradation mechanisms at the nanoscale under realistic battery operation environments.

#### 4.3.1 Electrode Configurations

The electrode configuration integrated in the electrochemical microchips plays a pivotal role in the overall performance of SiN_x_‐based closed liquid cells (Figure 3c). It not only governs electrochemical functionality, such as the uniformity and control of the electric field, but also influences mechanical robustness, liquid distribution, and compatibility with high‐resolution TEM imaging.

Over the years, two major electrode design paradigms have emerged, introduced by the two leading commercial suppliers of in situ TEM microchips: Hummingbird Scientific (Lacey, WA, USA) and Protochips (Morrisville, NC, USA) [156, 157].

Hummingbird Scientific introduced a comb‐shaped electrode layout, featuring three parallel platinum bar electrodes extending onto the SiN_x_ observation window (Figure 3c‐green). The terminal ends of these electrodes WE, RE, and CE are aligned co‐planarly and positioned within the imaging region. This configuration offers a relatively uniform electric field and allows simultaneous structural and electrochemical observation under the electron beam, making it particularly suitable for tracking dynamic electrochemical phenomena across extended areas. In contrast, Protochips proposed a radial electrode configuration, where a central, bar‐shaped working electrode with a rounded tip is located in the middle of the observation window (Figure 3c‐red) [157]. It is surrounded by arc‐shaped reference and counter electrodes, arranged concentrically. This design enhances the localization of the electric field, focusing the electrochemical interaction on the central region, and is especially well‐suited for studying single particles or localized reactions within confined zones. More recently, a novel electrode design has been proposed by Wei et al., introducing a configuration optimized for field homogeneity and mechanical stability in microscale liquid cells [158]. In this layout (Figure 3c‐yellow), a centrally positioned half‐round platinum WE is encircled by two concentric semicircular rings of varying width and radius, which serve as the counter and reference electrodes, respectively. All three electrodes are fabricated with a thickness of 50 nm. Compared to earlier designs, this configuration significantly improves the homogeneity of the electric field within the active imaging region. A key innovation of this design lies in the geometry of the observation window, which is maintained as a narrow rectangular aperture (∼30 nm wide) to minimize membrane bulging under high vacuum, which effectively increases the cell´s thickness. The half‐round working electrode is placed directly beneath the observation window, with only its upper edge exposed. This strategic placement aims to reduce mechanical stress on the fragile SiN_x_ membrane during operation, thereby lowering the risk of rupture and improving the overall durability of the device during in situ electrochemical cycling [158]. Using this configuration, Wei et al. investigated silver metal deposition mechanisms. They showed that the half‐round WE layout enabled uniform and mossy silver deposits with consistent lengths, in contrast to flat electrode configurations, which produced deposits with a random distribution. This study demonstrates that electrode geometry plays a critical role in controlling deposition morphology in microscale liquid‐cell experiments [158].

#### 4.3.2 Design of Spacers (Flow Management E‐Chip)

Spacers were among the earliest components introduced in closed LC technology, initially designed to control the liquid layer thickness inside the microchip (Figure 3c). Their primary role was to define the gap between the two SiN_x_ membranes, ensuring a suitable and consistent electrolyte thickness for both electrochemical operation and high‐resolution TEM imaging. In addition, spacers contributed to facilitating liquid flow and in some designs allowed for controlled mixing a feature useful in experiments requiring reactant homogenization or gradual reagent introduction. Spacer heights typically range from a few nanometers to several micrometers, depending on the desired liquid volume and resolution constraints [157].

Recent advancements have transformed the conventional spacer design into a more functional and dynamic flow management E‐chip (Protochips) [157]. This evolution involves integrating microfluidic bypass channels directly onto the chip, creating a hybrid architecture that combines diffusive and convective transport mechanisms. These on‐chip bypasses enable enhanced fluid circulation around the central nanochannel while preserving predominantly diffusive transport in the imaging region. This innovation brings several critical advantages: (i) Efficient removal of gas bubbles, preventing bubble accumulation that can terminate experiments prematurely. (ii) Controlled bubble formation and clearance, which can enhance image quality by displacing scattering media or clearing the field of view. (iii) Extended electrochemical potential ranges, enabled by better electrolyte replenishment and ion distribution. (iv) Improved hydrodynamic performance, with convective flow enhancements estimated to be 2–3 orders of magnitude higher than in traditional static or simple flow cell designs. This improved flow‐management architecture has significantly advanced in situ imaging of battery materials by providing more stable and reproducible experimental conditions. It was recently employed in an important study by Bejtka et al., who demonstrated how precise control of liquid flow is essential for mitigating gas‐bubble formation and ensuring efficient removal of gaseous by‐products. In their investigation of Zn metal electrodeposition, this design enabled real‐time tracking of the early‐stage morphological evolution of Zn with high resolution, even in a fully filled liquid cell [159].

#### 4.3.3 Extra Biasing Control Design

Another important design evolution that has further advanced MEMS‐based electrochemical chips involves the integration of multi‐functional capabilities, such as simultaneous electrochemical biasing and temperature control (Figure 3c). This new generation of EC chips includes additional electrodes or microheaters, enabling in situ heating and cooling within the liquid cell environment. By incorporating these thermal control elements alongside the standard three‐electrode electrochemical setup, researchers can now simulate realistic battery operating conditions, such as high‐temperature cycling or low‐temperature degradation scenarios, directly inside the TEM. This multifunctional configuration provides a powerful platform to investigate the coupled effects of temperature and electrochemical reactions on electrode behavior, interfacial stability, and degradation mechanisms. It represents a major step forward in bridging the gap between standard in situ experiments and the complex, real‐world conditions faced by LIB during practical use [157, 160, 161]. The dual regulation of heat and solvent conditions provides access to reaction pathways that cannot be captured under isothermal conditions. Recently, Khelfa et al. employed a temperature‐controlled liquid‐cell TEM to directly visualize the radiolysis‐driven formation of gold nanoparticles over a temperature range of 25°C–85 °C, revealing how temperature modulates nucleation kinetics, growth rates, and structural evolution at the atomic scale. This work demonstrates the significant advantage of combining thermal control with liquid‐phase TEM to uncover fundamental temperature‐dependent mechanisms in nanomaterial formation [160].

Strengths: SiN_x_‐based closed liquid cells offer a set of distinct advantages over earlier open or graphene‐based configurations. Their sealed, vacuum‐compatible design allows the use of real liquid electrolytes, closely replicating the operational environment of commercial lithium‐ion batteries. The integration of a three‐electrode configuration enables accurate and quantitative electrochemical control, while the electron‐transparent SiN_x_ membranes provide compatibility with high‐resolution TEM imaging under operando conditions. The system's dual operating modes static and flow offer versatility, with flow mode preventing local electrolyte depletion, removing gas bubbles, and maintaining a homogeneous chemical environment during cycling. Continuous design improvements, such as thinner membranes, optimized electrode geometries, and advanced flow management, have enhanced spatial resolution, stability, and electrochemical fidelity. Additionally, the incorporation of multifunctional capabilities, such as integrated heating and cooling, allows researchers to simulate realistic operating conditions, enabling detailed studies of coupled thermal‐electrochemical effects on battery materials.

Since their development, SiN_x_‐based liquid cells have represented a major advancement in understanding processes and mechanisms in LIBs, thanks to their ability to protect samples even for post‐in situ experiments.

## 5 Investigation of LIBs Using the Different TEM LC Designs: Achievements

The aforementioned LC designs have been employed extensively over the past decade for in situ TEM studies of LIBs. During this time, numerous investigations have demonstrated their ability to capture nanoscale structural and chemical dynamics in electrodes, electrolytes, and interfaces. These studies have provided valuable insights into processes such as degradation mechanisms, phase transformations, and interfacial evolution. Collectively, this body of work has significantly advanced our understanding of battery behavior and failure modes. Below, the key studies conducted using open cells, GLCs, and SiN_x_​‐based cells for the different components of LIBs, along with the main conclusions drawn from these investigations are summarized.

### 5.1 Open Liquid Cell

**FIGURE 4:**
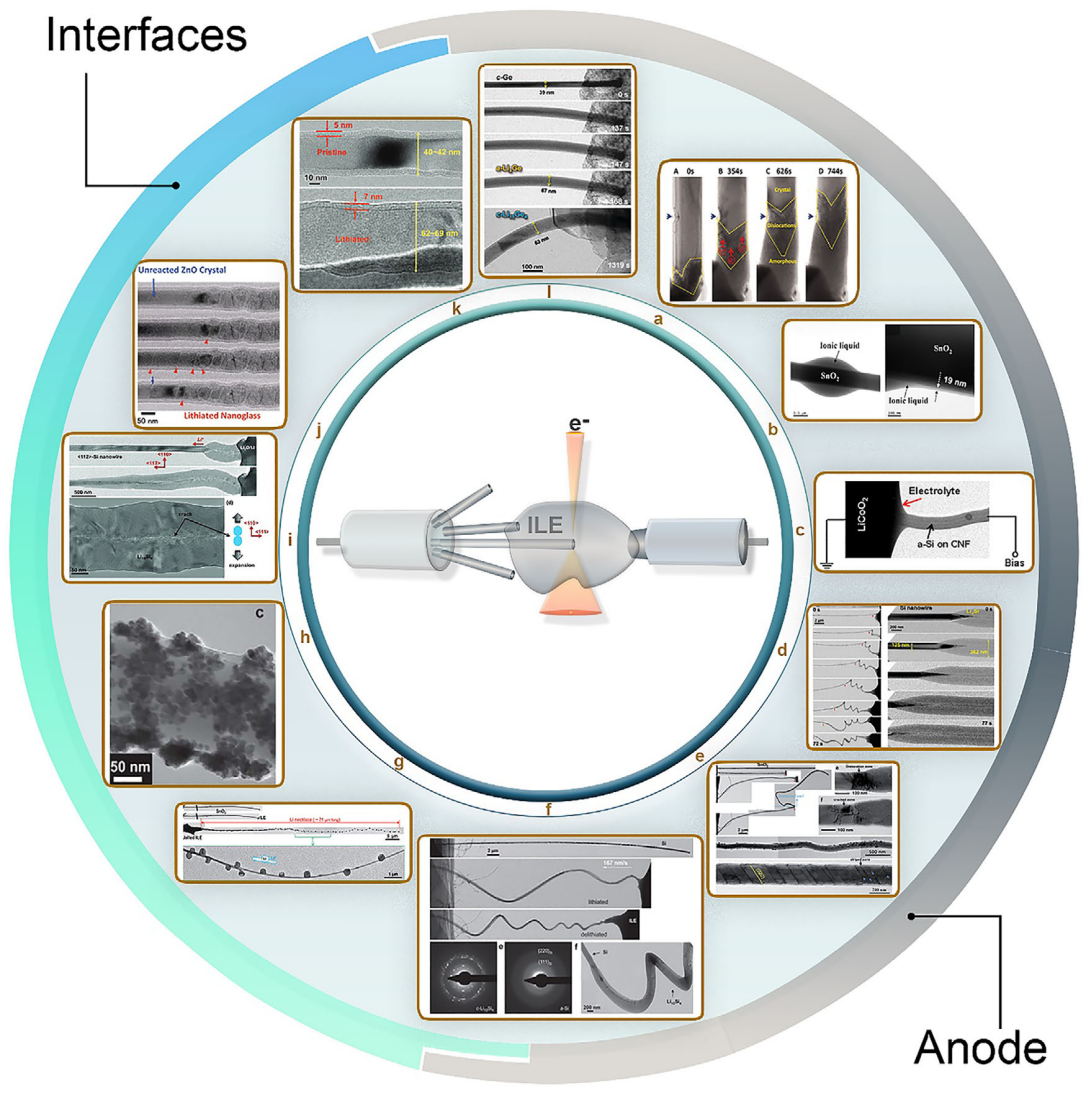
In situ TEM studies of anode materials and interfaces in LIBs using open‐cell configurations: (a) Copyright 2010, The American Association for the Advancement of Science. Lithiation of SnO_2_ nanowire: Reproduced with permission [142]. (b) Copyright 2011, The Materials Research Society. Behavior of SnO_2_ nanowire upon charging: Reproduced with permission [163]. (c) Copyright 2012, American Chemical Society. Structural evolution of silicon/carbon anode: Reproduced with permission [164]. (d) Copyright 2011, American Chemical Society. Lithiation of individual Si nanowire anodes: Reproduced with permission [165]. (e–h) Copyright 2011, Royal Society of Chemistry. Lithiation of SnO_2_ nanowires. Structural evolution of Si nanowires, Growth of Li dendrites on SnO_2_, and Ag nanoparticle plating on Si nanowires: Reproduced with permission [166]. (i) Copyright 2012, WILEY‐VCH. Studies of various nanowire and nanoparticle electrodes: Reproduced with permission [167]. (j) Copyright 2011, American Chemical Society. Cracking and nano‐amorphization of ZnO nanowires during lithiation: Reproduced with permission [168]. (k) Copyright 2011, American Chemical Society. Lithiation of Al nanowires; Reproduced with permission [169]. (l) Copyright 2011, American Chemical Society. Phase transformation in Ge nanowires: Reproduced with permission [170].

Pioneering the open‐cell approach to study battery materials, Huang et al., directly visualized in real time the lithiation of SnO_2_ nanowires during electrochemical charging using ILE (Figure 4a). A distinct reaction front was observed to propagate along the nanowire, causing swelling, elongation, and spiraling. This front contained a high density of dislocations appearing as a “cloud” indicating large in‐plane misfit stresses. Lithiation induced substantial volume expansion, plastic deformation, and eventual pulverization of the anode material [142].

The open‐cell configuration has since become a widely used platform for studying LIB materials, especially silicon‐based anodes and their electrolyte interfaces [162]. Silicon offers a much higher capacity than graphite, along with abundance, environmental safety, and low discharge voltage vs. Li/Li^+^. Wang et al. employed open‐cell TEM to track phase transformations and microstructural evolution in an amorphous silicon (a‐Si)‐coated carbon nanofiber (CNF) composite anode during cycling. High‐resolution real‐time imaging revealed that crystalline Li_15_Si_4_ formed from amorphous Li_x_Si via a spontaneous, congruent phase transition distinct from classical nucleation‐and‐growth. The a‐Si coating, strongly bonded to the CNF core, accommodated the large volume change reversibly without cracking in early cycles. With prolonged cycling, however, gradual degradation occurred, seen as progressive surface roughening (Figure 4b) [163]. In another study, Wang et al. examined a miniature battery with a single SnO_2_ nanowire anode and ILE. The thin liquid layer enabled ED analysis, which revealed the formation of an amorphous interphase, lithium oxide generation, and reversible Sn ↔ Li_x_Sn_y_ phase transformations (Figure 4c) [164].

Liu et al. (in 2011) combined HRTEM and ED to study the electrochemical lithiation of Si nanowire anodes. Carbon coating or heavy phosphorus doping enabled significantly higher charging rates compared to intrinsic Si. Real‐time observations showed that intrinsic Si nanowires lithiated slowly and incompletely, forming an amorphous Li_x_Si phase. In contrast, the modified nanowires rapidly formed crystalline Li_15_Si_4_ without fracturing, despite ∼300% volume expansion (Figure 4d) [165].

In a separate study, Liu et al. used both closed and open nanobattery cells to investigate multiple anode materials. The work provided local insights by showing that for SnO_2_ nanowires in ILE, lithiation initiated at the free end and propagated toward the root, causing swelling, elongation, spiraling, and dislocation nucleation at the moving front. Solid‐state amorphization resulted in elongated, twisted morphologies (Figure 4e) [166]. In the same study, C‐Si, P‐Si, and C+P‐Si nanowires were compared with intrinsic Si. For example, C‐Si nanowires converted to crystalline Li_15_Si_4_ upon lithiation and reverted to amorphous Si after delithiation (Figure 4f) [166]. This configuration also enabled direct imaging of Li dendrites growth as fibers on SnO_2_ nanowires, with LiF coatings (Figure 4g), as well as Ag nanoparticle plating on Si nanowires as SEI (Figure 4h) [166]. In a different study, Liu et al. extended open‐cell TEM to near‐atomic resolution studies of various nanowire and nanoparticle electrodes, building on their previous investigations of Si and SnO_2_ anodes. The study demonstrated that electrochemical reactions and degradation mechanisms are material‐specific, size‐dependent, and strongly influenced by geometry and composition. For example, Si nanowires exhibited highly anisotropic lithiation, with a critical diameter below which fracture was avoided, whereas larger Si nanowires developed surface cracking rather than central cracking. In contrast, Ge nanowires underwent nearly isotropic lithiation, forming a spongy network that could accommodate volume changes (Figure 4i) [167].

Beyond Si‐, Ge‐, and Sn‐based anodes, open liquid cells have been applied to other electrode materials. For example, Kushima et al. investigated the lithiation of single‐crystalline ZnO nanowires. HRTEM and ED revealed that the initial lithiation transformed the nanowires into a nanoglass composed of multiple glassy nanodomains. Real‐time imaging captured ∼70 nm nanocracks forming during partial lithiation, followed by rapid Li^+^ surface diffusion and solid‐state amorphization along the cracks. Subsequent crack merging produced glass–glass interfaces (Figure 4j) [168].

In another study, Liu et al. investigated Al nanowires coated with surface Al_2_O_3_ layers. In situ analysis revealed that lithiation always initiated within the oxide layer, forming a stable Li–Al–O glass tube a few nanometers thick. Only after this did the Al core undergo lithiation, transforming from single‐crystal Al to polycrystalline LiAl alloy, accompanied by significant volume expansion (Figure 4k) [169]. Finally, Liu et al. (in 2011) studied Ge nanowires and observed reversible nanoporosity formation during lithiation–delithiation cycling. Lithiation proceeded through two‐phase transformations: crystalline Ge → amorphous Li_x_Ge → crystalline Li_15_Ge_4_. Nanopores appeared only during delithiation, resulting from vacancy aggregation caused by lithium extraction (Figure 4l) [170]. These observations can directly guide battery materials design and optimization. For instance, real‐time visualization of large volume changes and anisotropic lithiation in Si and SnO_2_ nanowires identifies critical diameters and geometries that minimize fracture, informing the design of nanostructured electrodes with improved mechanical stability. Surface versus core lithiation, as observed in Al and Ge nanowires, can guide the development of protective coatings or artificial SEI layers to control stress distribution and suppress dendrite formation. Furthermore, insights into SEI nucleation, growth, and Li dendrite evolution enable rational optimization of electrolyte composition, additive selection, and deposition strategies to enhance interfacial stability and overall battery performance.

The open liquid cell TEM approach has provided unprecedented real‐time, high‐resolution insight into lithiation mechanisms, phase transformations, and degradation pathways in a variety of LIB electrode materials. It has revealed how composition, surface modification, geometry, and size critically influence lithiation kinetics and mechanical stability. Despite limitations such as beam effects, altered electrochemistry in nanoscale geometries, and non‐standard electrolytes, open‐cell TEM remains a powerful tool for correlating atomic structural evolution with electrochemical performance.

### 5.2 Graphene Liquid Cell

While open cells discussed above enable high‐resolution in situ imaging using ILEs, they are limited by the vacuum incompatibility of conventional electrolytes. GLCs overcome this by encapsulating a thin liquid layer between graphene sheets, allowing atomic‐resolution liquid‐phase TEM. Most GLCs lack electrical biasing, so the electron beam is often used to drive lithiation/delithiation, enabling observation of dynamic changes. Since 2012, GLCs have been widely applied to study LIBadvs73645 materials.

**FIGURE 5:**
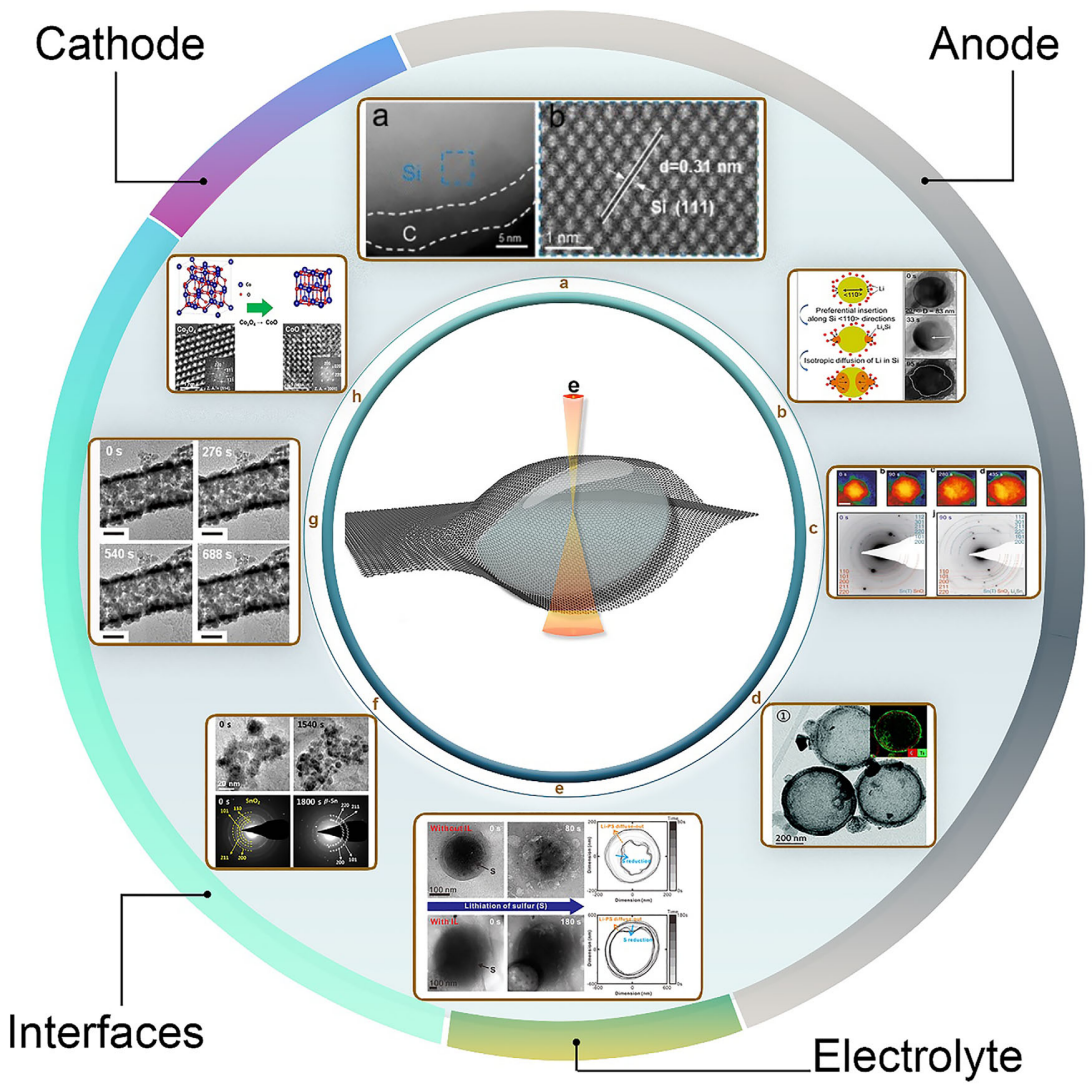
In situ TEM studies of LIBs using GLCs configurations: (a) Copyright 2024, Elsevier Ltd. Expansion behavior of Si nanoparticles and the influence of electrolyte composition: Reproduced with permission [171]. (b) Copyright 2014, American Chemical Society. Volume expansion of silicon nanomaterials during electrochemical lithiation: Reproduced with permission [172]. (c) Copyright 2019, Springer Nature. Phase and morphological evolution of single Sn nanoparticles during lithiation: Reproduced with permission [173]. (d) Copyright 2019, Royal Society of Chemistry. Growth mechanism of Li_2_S: Reproduced with permission [174]. (e) Copyright 2020, American Chemical Society. Morphological and phase evolution of sulfur nanoparticles during lithiation: Reproduced with permission [175]. (f) Copyright 2020, American Chemical Society. Conversion reaction and agglomeration dynamics of SnO_2_ nanoparticles in liquid electrolyte: Reproduced with permission [176]. (g) Copyright 2016, Elsevier Ltd. Formation of the SEI on SnO_2_ anode nanotubes: Reproduced with permission [177]. (h) Copyright 2014, American Chemical Society. Lithiation pathway of Co_3_O_4_ nanoparticles: Reproduced with permission [178].

For example, Yang et al. investigated the expansion behavior of Si NPs and the influence of electrolyte composition using GLCs. Real‐time atomic‐scale imaging revealed significant variations in the expansion rate of Si depending on the electrolyte environment, with chemical etching also observed during the process (Figure 5a) [171]. Yuk et al. were among the first to apply GLCs to LIB research, building on their earlier use for nanomaterials. They examined the volume expansion of silicon nanomaterials during electrochemical lithiation, simulated by electron‐beam irradiation to allow precise reaction control. ED analysis revealed anisotropic volume expansion of Si NPs, preferentially along the <110> crystallographic directions, a behavior attributed to the lower Li diffusion energy barrier at the Si‐electrolyte interface in these orientations. Additionally, they distinguished between the outer and inner regions of individual particles, highlighting differing expansion behaviors and offering deeper insights into lithiation‐induced nanoscale structural transformations (Figure 5b) [172]. Seo et al. employed GLCs to track the temporal phase and morphological evolution of single nanoparticles during lithiation under mechanical stress, revealing a strong coupling between stress and composition in lithium binary alloys. High‐resolution imaging showed the development of non‐uniform composition fields within Sn–SnO_2_ core–shell nanoparticles, with composition gradients directly proportional to the applied stress (Figure 5c) [173].

GLCs have also been applied to other Li‐based battery chemistries. Xu et al. investigated the growth mechanism of Li_2_S in high‐energy lithium sulfide batteries, demonstrating instantaneous nucleation followed by a transition from diffusion‐controlled to reaction‐limited growth kinetics, accompanied by a crystalline‐to‐amorphous phase transition (Figure 5d) [174]. Similarly, Seo et al. visualized lithium polysulfides (Li‐PS) and their suppression in liquid electrolytes, simultaneously tracking the formation and diffusion of Li‐PS alongside the morphological and phase evolution of sulfur nanoparticles during lithiation (Figure 5e) [175].

The anode–electrolyte interface has also been extensively studied using GLCs. Chang et al. leveraged the atomic‐scale resolution of GLCs to track the complete conversion reaction and agglomeration dynamics of SnO_2_ NPs in a liquid electrolyte. They observed sulfur nanoparticles nucleating on SnO_2_ surfaces and moving randomly to merge primarily through a multi‐step coalescence process involving rotation, neck formation, and merging. This study underscores the capability of atomic‐scale imaging in liquid electrolytes to reveal local mechanisms in LIBs, such as lithiation phenomena (Figure 5f) [176]. These observations can directly guide battery materials design and optimization. For example, understanding the nucleation, growth, and merging dynamics of Li_2_S or Li‐PS can inform strategies to suppress dendrite formation, control active material distribution, and design electrolyte formulations that enhance reaction uniformity and electrode stability in lithium–sulfur and other Li‐based batteries.

Cheong et al. applied GLCs to study the formation of the SEI on SnO_2_ anode nanotubes. Using electron‐beam irradiation to simulate lithiation, they observed lithium salt reduction and agglomerate formation on the nanotube surfaces, which gradually stabilized into a uniform SEI layer. The high spatial resolution enabled detailed tracking of SEI growth dynamics, confirming GLCs’ potential to monitor anode–electrolyte interface evolution with near‐atomic precision (Figure 5g) [177].

Cathode materials have also been studied using GLCs. Chang et al. examined the lithiation pathway of Co_3_O_4_ nanoparticles, achieving atomic‐scale resolution in liquid electrolyte during electrochemical lithiation. Real‐time imaging revealed the conversion of Co_3_O_4_ to CoO, confirming the thermodynamic favorability of this process (Figure 5h) [178].

Despite operating in static mode and lacking the capability for electrochemical biasing, GLCs have delivered unprecedented atomic‐resolution insights into electrochemical processes in real time. Their atomically thin graphene windows provide exceptional mechanical strength and chemical stability, enabling the encapsulation of liquid electrolytes while minimizing electron scattering. This configuration allows researchers to directly visualize nanoscale structural dynamics, ion transport, and reaction mechanisms with clarity that is not achievable using conventional liquid cells. While the inability to cycle materials electrochemically within GLCs limits their application for in situ battery operation, their unmatched spatial resolution continues to make them a powerful tool for fundamental studies of electrochemical phenomena at the atomic scale.

### 5.3 SiN_x_‐Based Liquid Cell

The SiN_x_‐based liquid cell is a versatile platform that enables real‐time observations of various LIB material dynamics in liquid electrolytes, closely mimicking the actual environment of commercial LIBs and processes that occur therein. The electrolyte composition can be tailored to experimental requirements, and different electrode materials both anodes and cathodes can be introduced via simple drop‐casting, although this method may result in high contact resistance. Additionally, the platform supports a wide range of electrochemical measurements, including cyclic voltammetry (CV), electrochemical impedance spectroscopy, and galvanostatic cycling.

Since its introduction in 2013, this design has become the most widely used for in situ and operando LIB studies. Unlike open cells (mainly for anodes) or GLCs (anodes and interfaces), the SiN_x_ cell offers unmatched versatility across electrodes, electrolytes, and interfaces.

**FIGURE 6:**
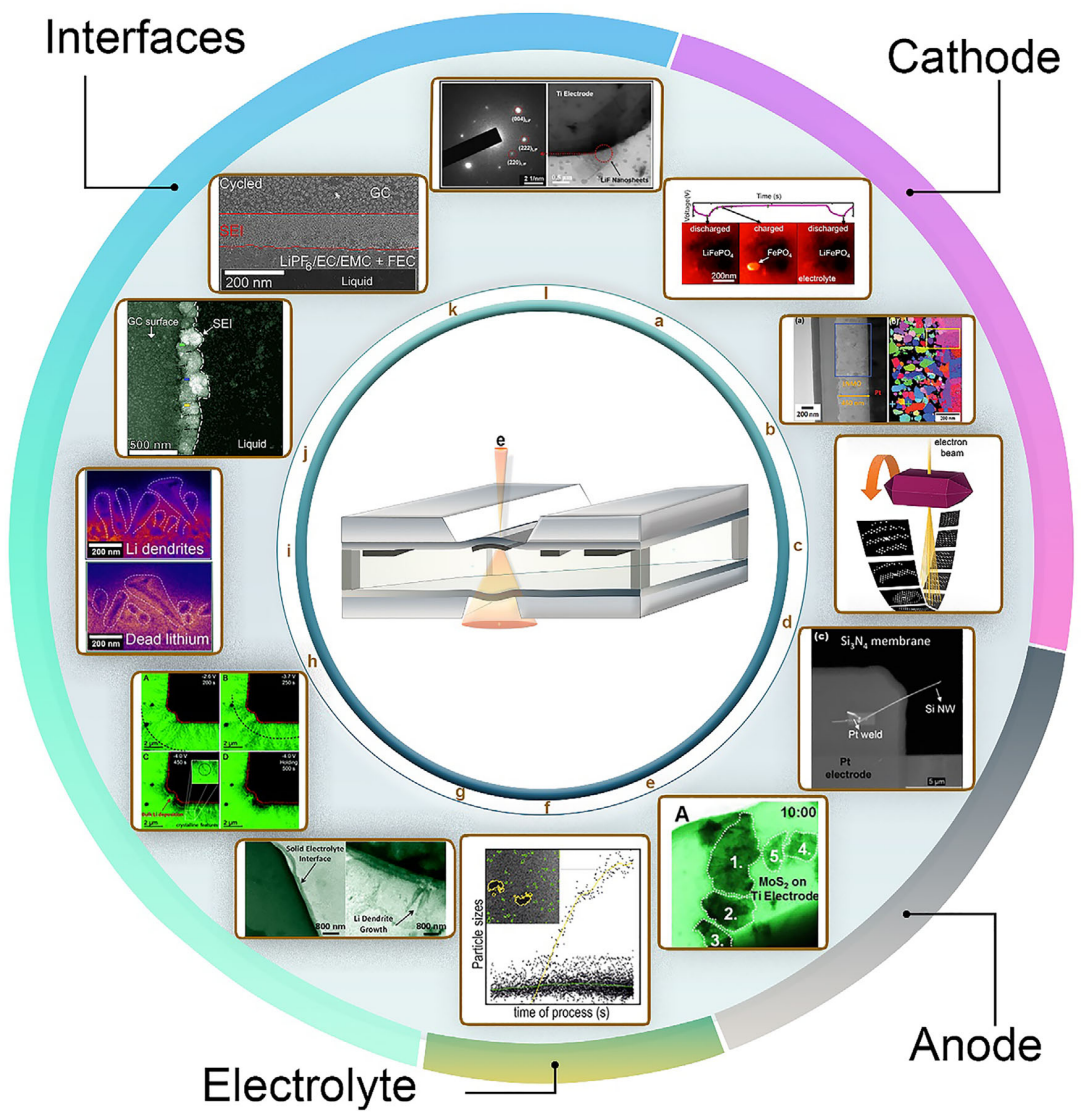
In situ TEM studies of anode materials and interfaces in LIBs using SiN_x_‐based liquid cell configurations: (a) Copyright 2014, American Chemical Society 2014. Lithiated/delithiated states of LFP: Reproduced with permission [179]. (b) Copyright 2021, WILEY‐VCH. Evolution of LMNO thin‐film cathode during charge–discharge cycling: Reproduced with permission [180]. (c) Copyright 2018, American Chemical Society. Li distribution in LFP during charge–discharge: Reproduced with permission [181]. (d) Copyright 2013, American Chemical Society. Lithiation/delithiation of fully submerged Si nanowire anodes. Reproduced with permission [182]. (e) Copyright 2015, American Chemical Society. Lithiation and delithiation of MoS_2_ nanosheets: Reproduced with permission [183]. (f) Copyright 2014, American Chemical Society. Degradation mechanisms of electrolytes: Reproduced with permission [184]. (g) Copyright 2014, American Chemical Society. Formation and transformation of the SEI: Reproduced with permission [185]. (h) Copyright 2014, Royal Society of Chemistry. Initial SEI morphology and Li deposition kinetics: Reproduced with permission [186]. (i) Copyright 2023, American Chemical Society. SEI growth mechanism: Reproduced with permission [187]. (j) Copyright 2024, Elsevier Ltd. Growth and dissolution of Li dendrites: Reproduced with permission [188]. (k) Copyright 2025, American Chemical Society. Role of additives in SEI growth: Reproduced with permission [189]. (l) Copyright 2022, Elsevier Inc. Formation of LiF nanosheets: Reproduced with permission [190].

On the cathode side, processes are particularly complex, with many occurring simultaneously and in parallel, requiring high spatial resolution to be disentangled. Despite these challenges, the SiN_x_‐based liquid cell has enabled significant advances. Holtz et al. employed this design to study LFP cathode nanomaterials during cycling. By applying low‐loss EELS, they overcame spatial resolution limitations imposed by the thick liquid medium and successfully tracked both solvated and intercalated Li ions, allowing the lithiation state of individual LFP particles to be determined (Figure 6a) [179]. In a related study, Bhatia et al. investigated the evolution of an LMNO thin‐film cathode during charge–discharge cycling in a liquid electrolyte. They observed structural and morphological changes associated with lithiation–delithiation, including void and crack formation, loss of contact with the current collector, and organic decomposition products. Furthermore, 4D‐STEM analysis revealed the onset of amorphization and a reduction in average grain size (Figure 6b) [180]. Extending these insights, Kurakulina et al. applied real‐time electron diffraction tomography to LFP cathode particles, a few hundred nanometers in size, and determined atomic coordinates and site occupancies, including Li distribution. This work demonstrated that the design enables to resolve structural information at the unit‐cell level during battery operation (Figure 6c) [181]. These insights provide guidance for rational design and optimization of cathode materials to mitigate structural degradation. Approaches may include developing cathode materials with enhanced resistance to amorphization, employing nanoscale architectures to reduce crack formation, applying protective coatings to stabilize particle morphology, and designing battery systems that incorporate real‐time diagnostics or self‐healing mechanisms. Such strategies, inspired by in situ LC‐TEM observations, can help preserve structural integrity, maintain electrochemical performance, and extend the operational lifetime of cathode materials.

Shifting focus from cathodes to anodes, the SiN_x_‐based cell has also been extensively employed to investigate lithiation–delithiation dynamics. In 2013 Gu et al. were the first to demonstrate its operando capability by directly observing lithiation and delithiation of fully submerged Si nanowire anodes. Unlike earlier open‐cell designs that lacked conformal liquid contact, this configuration replicated the liquid‐electrolyte environment of practical batteries. Their findings showed that while some behaviors such as lithiation‐induced amorphization were consistent with open‐cell studies, the closed cell provided new insights into electrode dynamics, including compositional changes, phase transformations, and the pronounced volume expansion associated with lithiation (Figure 6d) [182]. Building on this, Zeng et al. studied the lithiation and delithiation of MoS_2_ nanosheets on Ti electrodes. They reported irreversible decomposition upon discharge, leading to fragmentation of the nanosheets. Importantly, some nanosheets resisted decomposition, instead undergoing structural expansion and deformation, while an SEI layer simultaneously formed on the anode side (Figure 6e) [183].

In addition to electrodes, the SiN_x_‐based design has been applied to study electrolyte behavior under electrochemical cycling. Abellán et al. examined degradation mechanisms in a range of electrolyte solutions for LIBs, where localized electrochemical reactions were induced by the electron beam. Their work revealed that decomposition pathways of inorganic salts and solvents were complex yet consistent with known mechanisms (Figure 6f) [184].

Particularly notable progress has been achieved in investigating electrode–electrolyte interfaces. At the anode–electrolyte interface, the SiN_x_‐based liquid cell has enabled direct observation of SEI formation, Li dendrite growth, and dead Li formation. Zeng et al. examined the interface between an Au anode and a commercial electrolyte, successfully visualizing the formation and transformation of the SEI, as well as the nucleation and dissolution of Li dendrites (Figure 6g) [185]. Sacci et al. extended this work by directly visualizing initial SEI morphology and Li deposition kinetics, demonstrating that dendritic SEI morphologies form prior to Li metal deposition, and that after dissolution, residual Li remains on the electrode surface as dead Li (Figure 6h) [186]. Dachraoui et al. provided further insights, showing that SEI formation is a multistep process beginning with nanoparticle nucleation, followed by growth into island‐like domains, and ultimately evolving into a continuous mosaic‐structured layer composed of an inorganic‐rich inner and an organic‐rich outer region (Figure 6i) [187]. Using the same design, they elucidated Li dendrite growth and dissolution mechanisms, showing that dendrites develop through SEI‐controlled root‐ and tip‐driven growth, while dissolution is dictated by SEI evolution, ultimately contributing to dead Li formation (Figure 6j) [188]. Moreover, they demonstrated in another study that the additive FEC enhances the SEI and suppresses dead Li. Real‐time observations showed that FEC decomposition first forms a LiF‐rich nanoparticle‐based layer, followed by electrolyte decomposition that generates an organic layer binding to LiF. This layered structure promotes compact Li deposition and facilitates complete dissolution during discharge (Figure 6k) [189]. Such insights are valuable for electrolyte design, guiding the selection of additives like FEC to optimize the anode–electrolyte interface while controlling Li dendrite growth. In addition, LC‐TEM observations can inform electrolyte formulation and additive selection, allowing researchers to tailor electrolyte compositions for improved interface stability and battery performance. These studies can also guide anode structure and morphology design, enabling more homogeneous initial additive deposition, which helps suppress Li dendrites. Furthermore, the insights from the LiF‐rich initial layer can inspire the development of more efficient artificial SEI layers.

The cathode–electrolyte interface has also been extensively explored. Zhang et al. used the SiN_x_‐based platform to visualize the formation of LiF nanosheets on the CEI. They captured the dynamic deposition of LiF nanocrystals on positively charged Ti electrodes and tracked their structural evolution using ED. Notably, the nanocrystals exhibited merging, detachment, movement, and remarkable self‐healing behavior (Figure 6l) [190].

Overall, the SiN_x_‐based liquid cell has proven to be an exceptionally effective platform for investigating all key components of lithium‐ion batteries, including electrodes, electrolytes, and their interfaces. Its unique capability to closely replicate real electrochemical conditions allows researchers to observe dynamic processes in situ and operando, providing structural, morphological, and chemical insights that are otherwise difficult to achieve. This versatility makes it an indispensable tool for understanding the behavior of battery materials under realistic operating conditions.

Sections 4 and 5 discussed the history and technological evolution of the three LC‐TEM designs, highlighting their applications in studying degradation mechanisms in LIBs and clarifying the differences between them. Table 1 summarizes the main in situ liquid‐cell TEM designs for battery materials, comparing their liquid configuration, spatial resolution, cycling capability, key limitations, and typical applicability to various battery electrodes, electrolytes, and interfaces. While each design offers specific advantages for investigating particular processes in LIBs, direct quantitative comparison between the approaches is challenging, as the experimental setups are optimized for specific observation targets. Nonetheless, Table 1 provides a concise overview of the specifications of each setup.

**TABLE 1:** Comparison of the main in situ liquid‐cell TEM designs for battery materials.

Design	Liquid mode	Spatial resolution	Cycling configuration	Notes/Limitations	Battery sides accessible
**Open‐Cell**	‐Thin ‐Static	High	Potentiostat: two‐electrode	‐Limited to 1D/2D NMs ‐Low stability; strong ‐Strong beam‐induced artifacts	‐Interfaces ‐Anode (Figure 4)
**GLCs**	‐Thin ‐Static ‐Flow	High	Electron‐beam–triggered reactions (no EC control)	‐Mostly 2D NMs ‐Beam‐induced effects ‐Ultra‐fragile membrane	‐Anode ‐Cathode ‐Electrolyte ‐Interfaces (Figure 5)
**SiN_x_‐Cell**	‐Thick ‐Static ‐Flow	Low	Potentiostat: two‐ or three‐electrodes	‐Lower spatial resolution ‐Stable for long experiments ‐Fragile membrane	‐Anode ‐Cathode ‐Electrolyte ‐Interfaces (Figure 6)

### 5.4 Complementarity of TEM LC Designs for Multiscale Degradation Analysis

A unified understanding of LIB degradation requires capturing chemical and structural processes across multiple length scales, which no single LC‐TEM configuration can fully achieve. SiN_X_‐based liquid cells, which most closely mimic realistic electrochemical conditions with three or two electrode configurations and real electrolyte, enable tracking of mesoscale degradation phenomena such as SEI growth, particle cracking, and Li dendrite propagation. Their limited spatial resolution, however, prevents direct visualization of the very early stages of processes and the atomic‐scale interplay between processes. In this context, GLCs and open‐cell designs play a crucial complementary role. The ultrahigh spatial resolution of GLCs allows direct imaging of the initial steps of electrolyte decomposition, providing insight into the earliest SEI nucleation events and the reactive species involved. These observations complement the SEI growth mechanisms observed in SiN_X_‐based designs, filling the mechanistic gap at the atomic scale. For example, SiN_X_‐based designs have revealed a connection between SEI formation and Li dendrite morphology, showing that SEI growth influences dendrite growth and dissolution. However, the precise atomic‐scale interplay remains inaccessible due to limited resolution. Open‐cell designs, despite the absence of a real electrolyte, can reveal this atomic‐scale interaction, helping to understand dendrite stabilization or suppression and addressing the gaps left by SiN_X_‐based approaches. At the same time, SiN_X_‐based cell devices provide valuable information on the morphological and structural evolution of cathode materials during cycling. However, their spatial resolution limits access to particle‐level interactions with the electrolyte and the very first steps of CEI formation. These nanoscale details can be complemented by GLC observations, where encapsulating cathode materials with electrolyte enables ultrahigh‐resolution tracking of early interfacial chemistry and structural changes. Open‐cell configurations offer another level of complementarity. They enable high‐resolution studies of solid‐state lithiation processes, including anisotropic volume expansion, dislocation formation, fracture dynamics, and phase‐transition pathways within individual particles. These capabilities make open‐cell designs uniquely suited for probing the local mechanical–chemical coupling that drives cracking, pulverization, or phase‐instability mechanisms that refine and contextualize the mesoscale degradation features observed in SiN_X_‐based cells.

Together, these three LC‐TEM modalities; SiN_x_‐based cells, GLCs, and open cells form a coherent multiscale toolbox. By spanning device‐like electrochemical conditions, atomic‐scale chemical insight, and high‐resolution solid‐state dynamics, they collectively reveal degradation mechanisms from the earliest interfacial reactions to electrode‐level structural failure. This integrated perspective strengthens the interpretation of observations across Sections 5.1–5.3 and highlights the value of combining multiple designs to obtain a complete mechanistic picture.

Beyond acquiring S/TEM data in situ using these three designs, correlative approaches with in situ TEM can further enrich the dataset. For example, integrating optical excitation inside the TEM through dedicated light feedthroughs enables simultaneous optical and electron‐microscopy observations. Such correlative in situ measurements provide complementary information by combining wide‐field optical insights with high‐spatial‐resolution TEM data, thereby deepening the analysis of LIB materials [191]. Another promising correlative strategy is the combination of in situ TEM with Raman spectroscopy or laser‐based material modification. These methods complement standard EC‐LC‐TEM measurements by adding chemical and vibrational information. For instance, Raman spectroscopy can reveal characteristic vibrational signatures associated with carbonate decomposition or transition‐metal–oxygen bonding, helping to fill gaps in the interpretation of degradation pathways that are not accessible by TEM [192].

In addition, in situ EC‐LC‐TEM can benefit from holography approaches, such as gas electron holography, which enable the visualization and quantification of gas evolution in LIBs during operation [193].

Building on this multiscale perspective, the following section shifts from the capabilities of each design to a critical assessment of their remaining challenges. While Sections 5.1–5.3 highlighted the strengths and achievements of the three LC‐TEM configurations, each approach also presents intrinsic limitations that constrain the type and depth of degradation information that can be extracted. These limitations define the current bottlenecks in LC‐TEM research on LIBs and outline opportunities for future methodological development. The key limitations of each design in the context of LIB degradation studies are summarized below.

## 6 Limits of Open‐Cell in LIB Investigation

The open‐cell configuration, while widely used for nanoscale imaging (Figure 4), presents several technical challenges that limit its applicability for realistic LIB studies. Its exposed design confines the electrolyte to a static droplet, preventing fluid exchange and causing rapid local Li‐ion depletion during cycling. This restricts long‐term experiments and can introduce artefacts due to concentration gradients. The geometry favors quasi‐1D nanomaterials, such as nanorods or nanowires, reducing electrode–electrolyte contact to point‐like, highly confined interactions, which do not reflect realistic 2D interfaces. As a result, processes occurring on composite electrodes, cathodes, or complex morphologies such as SEI formation, surface reconstruction, and evolution of binder and conductive networks are difficult to capture.

The open‐cell design also exposes the system to the electron beam, making it highly susceptible to radiolysis, local heating, and undesired structural changes, which complicates reproducibility. Fragility of the setup and limited liquid coverage further restrict stable operation, while evaporation and contamination from the microscope vacuum can alter electrolyte composition, limiting experiment duration and the study of time‐dependent phenomena such as long‐term SEI evolution, continuous dendrite growth, or slow phase transformations.

Chemical representativeness is another key limitation. Open cells typically rely on non‐commercial electrolytes such as ionic liquids, whose viscosity, ionic conductivity, and wetting behavior differ from conventional LIB electrolytes. These differences can modify ion transport and interfacial chemistry, making it challenging to reliably observe processes such as electrolyte decomposition, solvent co‐intercalation, or dendrite formation under realistic conditions.

Despite these limitations, open cells remain valuable for proof‐of‐concept studies due to their simplicity, ease of fabrication, and capability for atomic‐ to nanoscale imaging. Nevertheless, their geometric, chemical, and operational constraints highlight the need for improved designs, more representative electrolytes, or hybrid approaches to achieve comprehensive characterization of practical LIB materials.

### 6.1 Limits of GLCs in LIBs Investigation

Graphene liquid cells offer atomic‐scale resolution due to their atomically thin membranes, but they present several technical challenges that limit their application to realistic LIB studies. The extreme fragility of the membranes makes fabrication and liquid sealing delicate, prone to rupture, and often low‐yield, reducing reproducibility and increasing experimental complexity and cost. Even in flow‐mode designs, the absence of robust sealing and integrated circuitry can lead to leakage and poor environmental control.

These structural limitations are compounded by the reliance on electron beam‐induced chemical reactions rather than true electrochemical biasing, which restricts controlled experiments. Despite the thinness of the graphene layers, beam‐induced effects such as radiolysis, heating, and electrolyte breakdown can occur, potentially distorting observations. In addition, chemical interactions between graphene and electrolyte species may alter the local environment, introducing artefacts not representative of real LIB conditions.

Another critical limitation is the lack of integrated functional electrodes suitable for electrochemical cycling. The fragility of the membranes, together with the inability to maintain a stable and well‐defined electrochemical environment, confines investigations primarily to model reactions or nanoparticle studies rather than realistic battery electrodes. Most setups operate in a static mode with extremely thin liquid layers (∼10—50 nm), restricting sample size and interface representativeness. Limited liquid volume, electrolyte evaporation, and gas bubble formation further constrain experiment duration and imaging quality. Beam sensitivity imposes additional restrictions on electron dose, forcing trade‐offs between resolution and experiment time.

As a result, key battery processes including lithiation/delithiation, SEI formation, dendrite growth, and cathode or interface evolution under cycling cannot be faithfully reproduced. While the approach provides unparalleled atomic‐scale resolution and valuable nanoscale insights, the combination of limited electrochemical control, minimal liquid volume, static operation, and sensitivity to beam and electrolyte effects restricts its ability to capture multiscale degradation processes in practical LIBs. Addressing these bottlenecks, for example, by developing robust electrodes, enhanced liquid management, or hybrid designs, would significantly expand the utility of these systems for in situ and operando LIB research.

### 6.2 Limits SiN_x_‐Based Cell in LIBs Investigation

SiN_x_‐based closed liquid cells are currently the most versatile platform for operando TEM studies of LIBs, enabling the investigation of all battery components including anodes, cathodes, interfaces, and electrolytes using commercial electrolytes and microbattery designs. Nevertheless, these cells present several technical limitations that constrain both experimental reliability and the depth of mechanistic insight.

The SiN_x_ membranes, although thicker and more robust than graphene, remain fragile and can rupture under high‐pressure differentials or mechanical stress. Several studies have examined the robustness and properties of SiN_x_ membranes used in TEM liquid cells. For example, Tiemand‐Lichtenberg et al. investigated the chemical and structural changes in commercial membranes over time, showing that electron beam exposure can induce significant transformations, converting SiN_x_ into silicon oxide in aqueous environments and generating gas release [194]. In another study, Koo et al. demonstrated that the average electron dose threshold required to rupture a SiN_x_ membrane in STEM mode ranges from 10^1^ ^0^ to 10^1^ ^1^ e^−^/Å^2^ for membranes 10—50 nm thick, and that the fracture toughness of an ultrathin SiN_x_ membrane corresponds to a pressure of approximately 13.55 GPa [195]. Such failures can abruptly terminate experiments and, in extreme cases, release liquid electrolyte into the TEM column, posing a potential risk to the microscope. Maintaining uniform liquid thickness is technically demanding, requiring contamination‐free assembly and precise MEMS fabrication, which adds complexity and cost. Integration of microfluidics or multifunctional capabilities, such as heating, further challenges mechanical stability.

Spatial resolution is inherently limited by the 10–50 nm thick membranes and the finite liquid layer, which increases electron scattering compared to graphene‐based cells. Electrode deposition and integration also present significant challenges. Commonly used materials, such as platinum or glassy carbon, can degrade under prolonged cycling or high bias, and far from representative of real battery anodes. Deposition techniques like drop‐casting may produce poor electrical contact and elevated resistance, compromising electrochemical measurements and the reliable monitoring of interface evolution. Additionally, the small observation window restricts statistical sampling of heterogeneous processes, limiting the generalizability of observations.

SiN_x_‐based cells are also sensitive to beam‐induced effects, including radiolysis, gas bubble formation, and unwanted chemical reactions, which can disrupt imaging and electrochemical stability. These limitations restrict experiment duration and the study of long‐term battery processes, such as electrolyte decomposition, phase transformations in electrodes, SEI formation, dendrite nucleation, and cathode surface reconstruction.

Overall, while SiN_x_‐based cells have significantly advanced operando TEM studies of LIBs, their current bottlenecks membrane fragility, limited spatial resolution, challenges in electrode integration, beam sensitivity, and difficulty controlling local electrochemical environments highlight the need for continued technical improvements to fully capture atomic‐scale battery phenomena under realistic cycling conditions.

## 7 Advances and Strategies to Overcome Bottlenecks

Unraveling the full complexity of LIB degradation is pivotal for guiding the design of next‐generation energy storage systems that combine extended lifetime, high performance, and uncompromised safety, particularly for demanding applications. A comprehensive understanding of these degradation pathways will not only enable the rational design of new electrode and electrolyte materials but also drive innovations in cell architecture and manufacturing processes.

**FIGURE 7:**
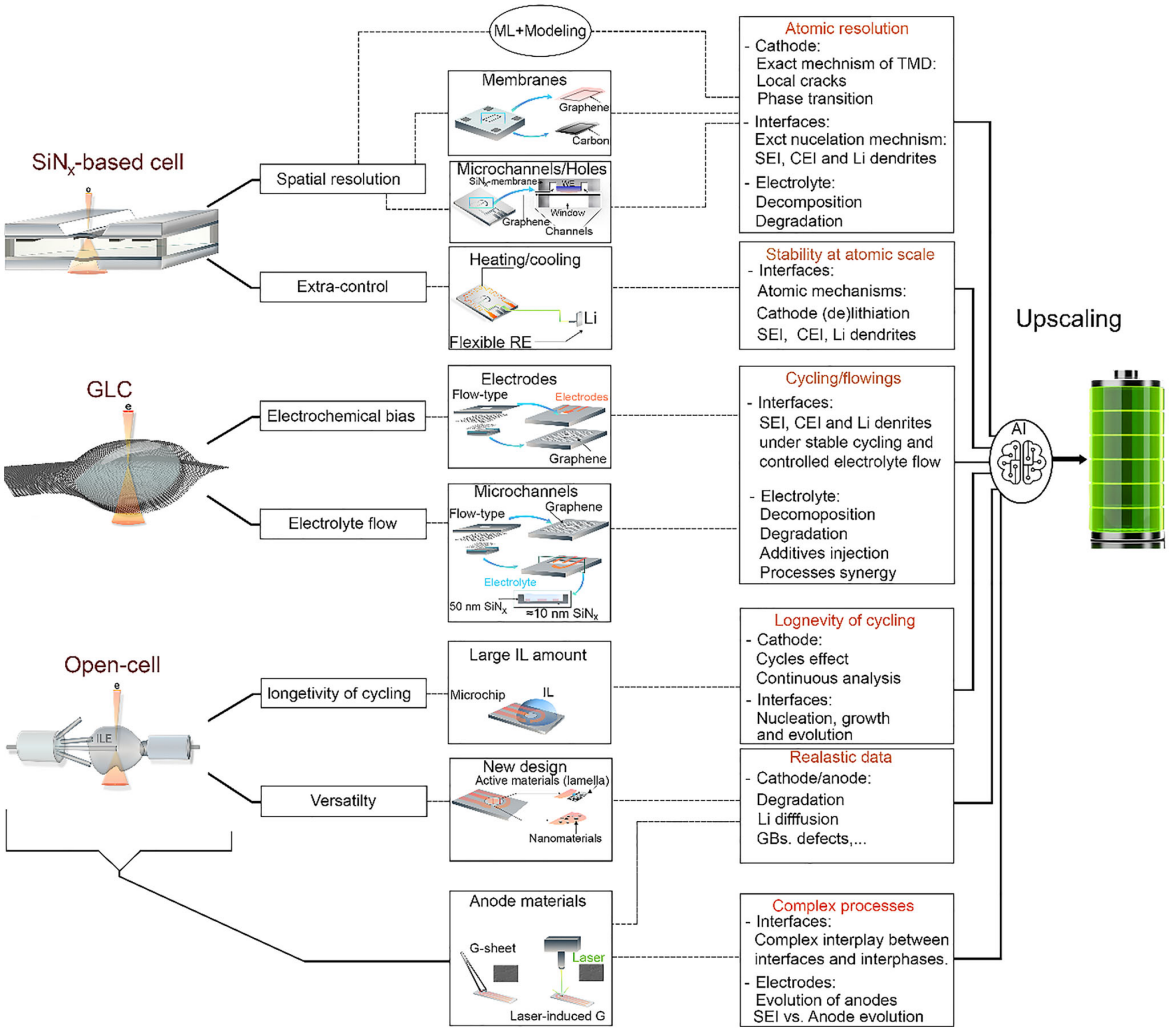
Schematic illustration of the pathways for advancing three liquid cell TEM designs; SiN_x_‐based cells, GLCs, and open‐cell configurations, toward deeper investigation of LIBs, and for upscaling operando nanoscale data to bridge the gap with real battery‐scale.

LC‐TEM has already provided unprecedented nanoscale insights into dynamic electrochemical processes as discussed in Section 5. Above, we discussed also the specific weaknesses of all three in situ TEM approaches, namely open cells, GLC, and SiN_x_‐based cell designs. Current bottlenecks in LC design, imaging, and electrochemical control still constrain the ability to probe critical degradation phenomena with the spatial, temporal, and electrochemical fidelity required. Addressing these limitations requires targeted strategies that span engineering advances in cell architecture, integration of novel materials for membranes and electrodes, optimization of electrochemical environments, and adoption of data‐driven approaches such as machine learning. Together, these innovations aim to deepen real‐time investigations of LIBs. Equally important is bridging the gap between nanoscale imaging in microcells and the behavior of practical batteries, for which we propose targeted approaches. Overcoming this gap will enable direct visualization of degradation mechanisms in their most fundamental form. The key directions are summarized in Figure 7.

### 7.1 Innovative Liquid Cell Design and Microfabrication Improvements

One promising direction toward realizing atomic‐scale resolution under realistic battery conditions including true electrolyte environments and electrochemical cycling is the advancement of SiN_x_‐based LCs. As outlined earlier, these designs enable the use of conventional battery electrolytes through encapsulation and flowing modes while maintaining electrode integrity for cycling. However, their relatively thick liquid layer and membranes limit atomic resolution imaging and analytics.

In contrast, GLCs offer what SiN_x_‐based cells lack: ultrathin graphene membranes and highly confined liquid thickness, achieved either through direct encapsulation or microchannel configurations, with recent flowing designs further enabling electrolyte exchange. A forward‐looking strategy is therefore to integrate graphene layers into SiN_x_‐based cells, combining the electrochemical versatility of SiN_x_ architectures with the superior spatial resolution afforded by graphene. Multiple approaches can be envisioned to realize such hybrid designs by leveraging the complementary strengths of both systems.

As illustrated in Figure 7, two types of hybrid membranes can be considered: graphene or ultra‐thin amorphous carbon films. Graphene is the most suitable due to its robustness and protective properties. However, direct integration of graphene as the top membrane on the EC chip is hindered by the presence of electrodes, which would create undesired electrical connections. A more practical approach is to use a graphene‐based bottom membrane either fully covering or partially reinforcing the SiN_x_ window to enhance mechanical stability, while simultaneously reducing the thickness of the upper SiN_x_ membrane. To further improve spatial resolution without compromising robustness, the upper membrane could be patterned with channels or holes, generating ultrathin regions (∼10 nm). Combined with graphene reinforcement, this architecture would enable atomic‐resolution imaging under realistic electrochemical conditions inside the TEM.

Beyond spatial resolution, it is also essential to probe battery materials and components including electrodes, electrolytes, and interfaces under realistic operating conditions such as elevated or low temperatures. To achieve this, temperature control should be integrated alongside electrochemical operation. This can be realized by incorporating dedicated heating/cooling electrodes, as illustrated in Figure 7. Notably, the graphene membrane proposed for the bottom chip could also serve as an efficient heating platform, providing both mechanical support and localized thermal control.

GLCs, as previously discussed, have evolved significantly from static to flowing and even mixing modes, offering unprecedented spatial resolution. However, for battery studies, the ultimate goal remains the integration of full electrochemical control into these designs. This is particularly challenging due to the fragile nature of graphene sheets. As shown in Figure 7‐GLC, one possible approach is to exploit flow‐mode GLCs by engineering the top membrane: replacing graphene with carbon or an ultrathin (∼30 nm) patterned SiN_x_ membrane. For a flowing operation, the same principle can be applied by incorporating channels into the top membrane to ensure continuous electrolyte delivery. Such developments would combine electrochemical control with ultrathin membranes and confined liquid, bringing the field closer to the long‐standing goal of achieving atomic‐scale resolution while cycling batteries under realistic conditions inside the TEM.

### 7.2 Leveraging Machine Learning to Enhance Resolution and Data Analysis

Machine learning has emerged as a powerful tool to address several challenges inherent in liquid cell TEM studies of LIBs. A major bottleneck in these studies is electron beam–induced damage and noise, which obscure subtle nanoscale features and limit experiment duration. ML algorithms, including advanced deep learning models, are being implemented to denoise images and videos in real time, substantially improving the signal‐to‐noise ratio without increasing the electron dose. This enables longer observations and acquisition of higher‐quality data.

Beyond denoising, ML‐based image analysis can automate the detection and tracking of dynamic processes such as dendrite growth, SEI formation, and phase transformations, allowing the extraction of quantitative metrics that were previously impractical to measure manually. Generative approaches, such as generative adversarial networks (GANs), also show promise in reconstructing high‐resolution images from lower‐quality raw data, effectively overcoming some intrinsic spatial limitations of liquid cell environments. Furthermore, ML approaches might be able to differentiate beam‐induced artifacts from genuine electrochemical phenomena, thereby enhancing the reliability and interpretability of operando TEM results.

A particularly promising direction is using ML to bridge the gap between nanoscale observations and atomic‐scale dynamics. Although liquid environments often restrict direct atomic resolution imaging, ML models can infer hidden structural information by integrating nanoscale TEM data with physical simulations. Coupling ML with phase‐field modeling (PFM) is especially powerful, as PFM requires spatially resolved input such as Li distribution maps obtained from dark‐field TEM (DF‐TEM) or energy‐filtered TEM (EFTEM). These datasets provide the necessary nanoscale information to track lithium‐ion migration within single cathode particles or interfacial regions, enabling reconstruction of probable Li diffusion pathways during charge–discharge. Advanced architectures including convolutional neural networks for feature extraction, recurrent neural networks or transformers for capturing temporal evolution, and physics‐informed neural networks (PINNs) for embedding electrochemical and diffusion constraints further strengthen this approach. By combining DF‐TEM/EFTEM‐driven PFM with ex situ atomic‐resolution datasets, ML can interpolate as well as extrapolate atomic dynamics across multiple electrode regions and interfaces, ultimately pushing liquid cell TEM analysis toward resolving true atomic‐scale behavior [196, 197, 198, 199, 200, 201, 202, 203].

### 7.3 Development of Advanced Nanomaterials and Electrode Architectures for Liquid Cell Studies

To maximize the potential of LC‐TEM, developing tailored nanomaterials and electrode architectures specifically optimized for in situ characterization could offer significant benefits. Designing electrodes with nanostructured geometries such as arrays of nanowires, nanosheets, or porous frameworks facilitates uniform electrolyte access and reduces interfacial resistance, thereby enhancing both electrochemical performance and imaging quality. These architectures also provide well‐defined model systems for directly correlating nanoscale structural evolution with electrochemical behavior.

Surface engineering further improves the fidelity of LC‐TEM studies. For example, protective or functional coatings that stabilize SEI formation or suppress dendrite growth enable more representative and reproducible observations of battery processes. Incorporating conductive and chemically inert support layers beneath active materials helps mitigate beam damage while improving electron transparency. Additionally, hybrid electrolyte systems and thin‐film electrode materials compatible with LC platforms are being explored to better mimic next‐generation battery chemistries. Collectively, these advances enable more accurate operando investigations, offering deeper insights into degradation mechanisms and guiding the design of improved LIB systems.

Despite significant progress, most LC‐TEM studies still employ SiN_x_‐based devices in which the working electrodes are typically platinum, glassy carbon, or gold. While these materials are convenient for device fabrication and imaging, they do not represent the actual anode materials used in commercial lithium‐ion batteries, which are predominantly graphite or other carbon‐based electrodes. This mismatch restricts the relevance of interfacial studies, particularly for investigating SEI growth, Li dendrite nucleation, and Li‐ion diffusion mechanisms under realistic conditions. To better mimic true battery environments, it is therefore essential to integrate real anode materials into LC‐TEM platforms. In this regard, graphene has emerged as a highly promising candidate due to its structural similarity to graphite, combined with excellent conductivity, chemical stability, and compatibility with in situ TEM. Recent advances now enable the deposition of graphene directly onto SiN_x_ chips, either through simple transfer of graphene sheets onto Pt or glassy carbon electrodes, or through more sophisticated approaches such as laser‐induced graphene deposition, which offers precise and efficient electrode fabrication (Figure 7). These developments represent an important step toward more realistic operando studies of battery interfaces using LC‐TEM [204, 205, 206, 207, 208, 209, 210, 211, 212, 213].

### 7.4 Upscaling: Bridging Nanoscale Observations to Real Battery Scale

Materials and battery configurations studied in situ using TEM are often far from representative of commercial systems. Simplifications such as flat electrode geometries, limited electrolyte volumes, higher current densities, and the use of model electrodes (e.g., glassy carbon, platinum, or gold) differ substantially from practical cells. These discrepancies can strongly influence reaction kinetics, SEI formation, dendrite growth, and degradation pathways, thereby limiting the direct applicability of TEM observations to real‐world batteries.

Upscaling strategies aim to overcome this gap by integrating LC‐TEM data with multiscale modeling and ML. This combined approach enables the translation of insights gained from simplified local systems into the complex, 3D, and heterogeneous electrode–electrolyte environments found in commercial batteries. For instance, SEI growth at a model GC‐electrolyte interface can be quantitatively tracked with LC‐TEM. ML algorithms can then extract the underlying mechanisms such as nucleation site distribution, growth kinetics, and compositional evolution, and extrapolate them to realistic battery electrodes, where SEI formation occurs simultaneously on numerous particles in a dynamic 3D network.

By linking nanoscale in situ observations with mesoscale and continuum models, predictive frameworks can be established to capture interfacial dynamics, electrolyte decomposition, and long‐term degradation mechanisms that cannot be directly visualized at atomic resolution during operando cycling. Moreover, these upscaling strategies provide valuable design guidelines: they can identify critical structural and chemical descriptors of degradation, suggest optimized electrode architectures, and bridge fundamental mechanistic insights with practical battery performance.

Several ML strategies enable this translation:

- PINNs encode electrochemical laws and diffusion physics, allowing predictions under unobserved conditions and complex geometries [205].
- Graph neural networks capture particle connectivity and heterogeneity in real electrodes, enabling simulation of Li transport and dendrite propagation across 3D networks [202].
- Generative models (GANs, VAEs) reconstruct high‐resolution or atomic‐scale features from lower‐resolution LC‐TEM data, bridging simple interfaces to realistic, complex systems [214].
- Transfer learning leverages models trained on simple, well‐controlled experiments and adapts them to more complex materials, electrolyte compositions, or cycling conditions [198].
- Reinforcement learning and surrogate modeling iteratively optimize electrode designs or cycling protocols by connecting local LC‐TEM observations with predicted full‐cell behavior [203].

Coupled with PFM, ML‐driven upscaling allows simulation of Li‐ion diffusion, SEI evolution, and interfacial dynamics in realistic 3D electrodes. By integrating atomic‐ and nanoscale LC‐TEM insights with ML and PFM, it becomes possible to predict long‐term cycling behavior, stability limits, and failure modes in commercial devices.

As illustrated in Figure 7, upscaling relies on three pillars: (i) atomic‐ and nanoscale TEM insights, (ii) realistic electrode materials and architectures, and (iii) ML‐driven multiscale modeling. Altogether, this approach transforms LC‐TEM from a purely diagnostic technique into a predictive platform that guides the rational design of commercial LIBs.

## 8 Beyond Lithium‐Ion Battery Materials

The strategies outlined in Section 7 to overcome current limitations in LC‐TEM studies of LIB materials provide a conceptual framework with broad implications for materials research. While these solutions are still prospective, their potential to enhance operando nanoscale characterization positions LC‐TEM as a versatile tool for investigating a wide spectrum of electrochemical and functional materials. By addressing fundamental bottlenecks in spatial resolution, electrochemical control, and data interpretation, these approaches can transform the way dynamic materials processes are visualized and understood.

Other battery chemistries including sodium‐, zinc‐, magnesium‐, and lithium–sulfur systems face challenges analogous to LIBs, such as dendrite formation, interphase evolution, electrode degradation, and electrolyte decomposition. Similar instability mechanisms have been reported in sodium and zinc metal anodes, where dendrite propagation and interfacial gas evolution critically affect cycling reversibility [215, 216]. Improvements in liquid cell design enabling thinner liquid layers, enhanced biasing control, and greater imaging stability combined with machine learning–assisted characterization and upscaling from microcells to full cells, promise to significantly expand LC‐TEM capabilities across these energy systems. These advances can provide mechanistic insights into otherwise inaccessible processes, guiding the rational design of safer, more efficient, and longer‐lasting energy storage materials.

The benefits of enhanced LC designs can be extended to other electrochemical systems, including fuel cells and photoelectrochemical materials. LC‐TEM enables direct operando visualization of catalyst restructuring, electrode corrosion, ion transport, and light‐driven redox reactions, yielding mechanistic insights that are inaccessible by conventional methods. Combining advanced LC designs including thin membranes, controlled liquid environments, and precise biasing with ML‐assisted analysis and multiscale upscaling allows higher spatial resolution, better control over reaction conditions, and real‐time correlation of nanoscale dynamics with macroscopic performance. Collectively, these capabilities overcome long‐standing limitations of in situ TEM and open new opportunities for investigating a broad range of energy‐conversion and functional materials [217, 218].

Importantly, the strategies proposed here can establish a generalizable and scalable platform for materials research. By integrating innovative liquid cell architectures, machine learning, and multiscale modeling, LC‐TEM can interrogate chemical and structural evolution, interfacial phenomena, and degradation mechanisms across diverse functional systems. While motivated by LIB studies, these approaches provide a roadmap for extending operando nanoscale investigations to virtually any functional material in liquid environments, enabling predictive insights and accelerating the development of next‐generation energy storage, conversion, and catalytic materials.

Each electrochemical energy storage system, including LIBs, sodium‐, zinc‐, magnesium‐, and lithium–sulfur batteries, presents specific challenges that require tailored LC‐TEM conditions. These challenges arise from the distinct physicochemical properties of electrolytes and electrode structures. For instance, some electrolytes are highly air‐ or moisture‐sensitive, and certain electrode morphologies may be more fragile or require different ionic conductivities. Such constraints affect membrane selection, liquid thickness, and electrochemical control. Taking these factors into account in cell design such as using exchangeable tubing systems or microchip materials specifically chosen for compatibility with each battery chemistry can make LC‐TEM configurations more versatile and adaptable across different energy storage systems.

## 9 Conclusions

LIBs have revolutionized modern technology by enabling portable electronics and replacing fossil fuels in electric vehicles, thereby accelerating the transition toward green energy. Meeting future demands including large‐scale energy grids and electric aircraft will require even higher performance and reliability, yet intrinsic degradation processes continue to limit battery efficiency and lifespan. Understanding these mechanisms has challenged researchers for decades, with in situ techniques, particularly LC‐TEM, proving among the most powerful tools. Although three main LC‐TEM cell designs have been developed, each faces bottlenecks that hinder deeper insights into battery processes.

This review summarizes LIB degradation mechanisms, highlights TEM capabilities, examines existing cell designs, evaluates their achievements and limitations, and proposes strategies to overcome these challenges. By providing a comprehensive yet accessible overview, it aims to engage readers from diverse backgrounds in both LIBs and in situ TEM, fostering new ideas and guiding future research. Notably, it also outlines solutions to bridge the gap between localized in situ analysis and the complex environment of real batteries at millimeter‐to‐centimeter scales. Importantly, the strategies presented here suggest a broader role for LC‐TEM in materials research. Methodological innovations developed to address LIB complexities can be leveraged to study advanced materials across batteries, fuel cells, catalysis, and corrosion. By extending these solutions to diverse systems, LC‐TEM has the potential to become a transformative platform, linking atomic‐ and nanoscale processes to macroscopic performance and informing the rational design of advanced materials with unprecedented precision and impact.

## Author Contributions

W.D. and R.E. wrote the manuscript. Both authors discussed and commented on the manuscript.

## Conflicts of Interest

The authors declare no conflict of interest.

## Data Availability

The data that support the findings of this study are available from the corresponding author upon reasonable request.
